# Grapevine bacterial communities display compartment-specific dynamics over space and time within the Central Valley of California

**DOI:** 10.1186/s40793-023-00539-0

**Published:** 2023-11-23

**Authors:** Joel F. Swift, Zoë Migicovsky, Grace E. Trello, Allison J. Miller

**Affiliations:** 1 Department of Biology, Saint Louis University, 3507 Laclede Avenue, St. Louis, MO 63103 USA; 2 Donald Danforth Plant Science Center, 975 North Warson Road, St. Louis, MO 63132 USA; 3 Department of Plant, Food and Environmental Sciences, Faculty of Agriculture, Dalhousie University, Truro, NS B2N 5E3 Canada; 4 Present Address: Kansas Biological Survey and Center for Ecological Research, University of Kansas, Lawrence, KS 66045 USA; 5 Present Address: Department of Biology, Acadia University, Wolfville, NS B4P 2R6 Canada

**Keywords:** Microbiome, Grapevine, Grafting, California, Central Valley, Amplicon sequencing

## Abstract

**Background:**

Plant organs (compartments) host distinct microbiota which shift in response to variation in both development and climate. Grapevines are woody perennial crops that are clonally propagated and cultivated across vast geographic areas, and as such, their microbial communities may also reflect site-specific influences. These site-specific influences along with microbial differences across sites compose ‘terroir’, the environmental influence on wine produced in a given region. Commercial grapevines are typically composed of a genetically distinct root (rootstock) grafted to a shoot system (scion) which adds an additional layer of complexity via genome-to-genome interactions.

**Results:**

To understand spatial and temporal patterns of bacterial diversity in grafted grapevines, we used 16S rRNA amplicon sequencing to quantify soil and compartment microbiota (berries, leaves, and roots) for grafted grapevines in commercial vineyards across three counties in the Central Valley of California over two successive growing seasons. Community composition revealed compartment-specific dynamics. Roots assembled site-specific bacterial communities that reflected rootstock genotype and environment influences, whereas bacterial communities of leaves and berries displayed associations with time.

**Conclusions:**

These results provide further evidence of a microbial terroir within the grapevine root systems but also reveal that the microbiota of above-ground compartments are only weakly associated with the local soil microbiome in the Central Valley of California.

**Supplementary Information:**

The online version contains supplementary material available at 10.1186/s40793-023-00539-0.

## Background

Plants form associations with microorganisms in different compartments across the plant body, including roots, leaves, and fruits. Microbiota vary strongly across compartments due to differences in physical and chemical properties, resource availability, and environmental factors [1–5]. Host genetics also play a role in dictating compartment microbiota. For example, root exudate profiles and plant immune responses are often genotype-specific and contribute to shaping the plant microbiome [6–10]. Some associations formed between plants and microorganisms are beneficial to plant survival as microorganisms are able to promote growth and confer resistance to many biotic and abiotic stressors [11–13]. Thus, research has focused on understanding factors that shape a plant’s microbiota, including what stage of development microbial associations form and how stable they are over space and time.

Many biogeographical studies have shown strong patterning of plant compartment microbiota by geographic location or site [22–25], owing to both biotic and abiotic factors. Soil serves as the primary reservoir of microorganisms that interact with the below-ground portion of plants [14–19], occurring through direct recruitment from the soil to the root surface and internal tissues. Above-ground plant compartments (*e.g.,* leaves and fruits) interact with soil microbes via internal transport between tissues, but also receive many microbial colonizers by deposition via rainfall and wind dispersal [20, 21]. Vegetation patterns and cover shape soil microbiota along with abiotic soil properties, including texture, pH, and chemical composition [22–27]. Agricultural plant species are grown across wide geographic areas, and are subject to varying management regimes [28]. This makes them ideal for investigating site-specific influences on the microbiota of different compartments of the plant.

Plant microbiomes are dynamic [19, 29–32] and change over development [33, 34]. In annual plants, a two-stage model of microbiome assembly has been proposed [31]. Following seed germination, seed endophytes [35] and microorganisms in close proximity colonize root tissues to form a juvenile microbiome; many of these early colonizers are displaced, via competition or host selection, over time to form a more stable adult microbiome. This shift to an adult microbiome is hypothesized to be the result of variation in root exudate composition during a plant’s life cycle [36, 37], which can change rapidly [38]. Perennial plants have an extended life cycle and, for many temperate species, periods of dormancy, increasing their complexity. In the herbaceous perennial *Arabis alpina,* the bacterial community composition of the root endosphere shifted with residence time in the soil, enriching for Proteobacteria and while Bacteroidetes was depleted [39]. For the perennial mustard, *Boechera stricta*, a study utilizing multiple field sites found that the root microbiota shifted over time, with bacterial diversity of the root decreasing over the four years of the experiment, whereas leaf microbiota were less responsive to the age of the plant and more responsive to the plant genotype [40]. These data demonstrate compartment-specific patterning between leaves and roots. It remains to be seen whether these patterns in short lived herbaceous perennial plants is generalizable to perennial crop species, which allocate considerably more resources to harvestable organs than wild species [41] or to woody perennials, which have substantially longer lifespans [42, 43].

Woody perennial crops offer an excellent system to investigate how microbiota of plant compartments differ among genotypes, across sites, over the course of a growing season, and from one year to the next. Grapevines (*Vitis* spp. L.) are long-lived woody perennials (> 20 production years), in which cultivated varieties (cultivars) are clonally propagated and grown across different regions. Studies have found biogeographical patterning to the microbiome of vineyard soils [44, 45] and differences in grapevine microbiota across sites, often termed microbial terroir [16, 46–48]. Previous microbiome studies profiling grapevine berries and musts across both regional and local scales have found signatures of geographic location on berry bacterial and fungal communities, with some evidence these microbial communities contribute to fermentation and wine characteristics [49]. A study by Zarraonaindia et al*.* [16], profling of compartments of both the root and shoot system (*i.e.*, flowers, berries, leaves) found that vineyard soil served as the primary reservoir of plant-associated bacteria and that intra-vineyard heterogeneity in soil edaphic factors outweighed larger biogeographic trends. Given the dynamic nature of plant microbiomes over time, we sought to further investigate biogeographical patterns in grapevine microbiota across compartments by designing our sampling to capture both intra-seasonal (multiple timepoints within a season) and annual temporal components. In a similar study on grapevine fungal microbiota, compartments displayed contrasting responses based on vineyard location and growing season [32]. Fungal diversity of the root varied strongly by vineyard, while all other compartments (root, leaf, flower, berry) varied across the growing season according to developmental stage (flowering, fruit set, veraison, harvest). These data suggest that the grapevine microbiome is dynamic over time, and that patterns of fungal diversity vary across the vine.

An additional factor affecting the grapevine microbiome is grafting, a common horticultural technique used in viticulture which joins different genotypes together. Grafting joins the rootstock (root system and lower stem) and the scion (including the upper stem, leaves, flowers, fruits) together [50, 51]. Whereas scion genotype is selected primarily based on the grape cultivar desired for fruit quality, rootstock genotypes in grapevines are selected based on their ability to confer resistance to pest and pathogens [50], but have also been utilized to control scion vigor and yield [52]. Recent work has expanded current understanding of interactions between grafted rootstock and scion [53–58], and has demonstrated that root microbiome diversity and composition is dependent on not only the rootstock genotype [59–62], but also the interaction with the scion genotype [63, 64]. For example, across various species, scion genotypes have been shown to alter root system architecture [65], biomass allocation patterns [66], root transcriptomic responses to nutrient limitation [67], and root organic acid concentration [68]. In addition, elemental composition of scion tissues has been shown to be dependent on rootstock genotype [54–57]. Less is known about how rootstock genotype influences the microbiota of the leaves and berries in grafted grapevines. Thus, we sought to use grafting to explore interactions between the soil microbiome, environment, and the genomes of the host, both rootstock and scion, and their influence on the microbiota of different compartments of the vine.

Our work investigated the bacterial communities of grapevine compartments of multiple rootstock/scion combinations across growing seasons, in geographically distinct vineyards, and over multiple years. We hypothesize that the microbiota of each plant compartment reflects, at least in part, the geographic location in which the host plant is grown. Clonally propagated perennial plants offer a unique opportunity to test this hypothesis as the host genotype can be planted and maintained over time across a wide range of sites to investigate site-specific influences on the microbiota of different compartments of the plant. To do this, we characterized bacterial communities in compartments of rootstock/scion combinations replicated across three commercial vineyards in the Central Valley of California, over the course of two growing seasons. Our objectives were to (1) assess soil structure, elemental composition, and microbiome across sites, (2) characterize seasonal and yearly patterns of bacterial communities across vine compartments, and (3) determine relative contributions of compartment, site, rootstock genotype, and scion genotype to patterns of microorganism community composition across vine compartments.

## Methods

### Experimental design and sampling

**Fig. 1:**
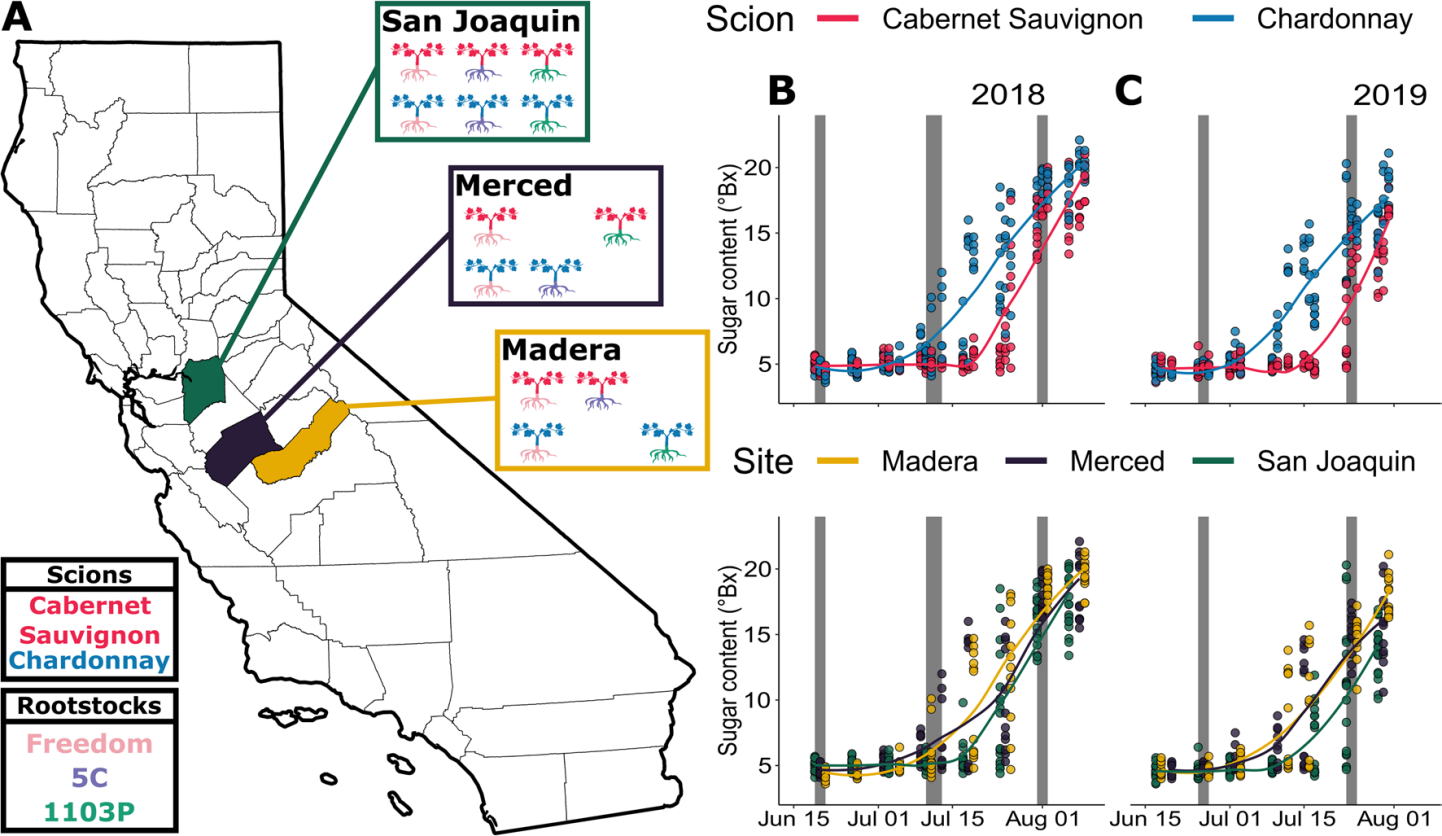
Multiple scion/rootstock combinations were sampled across vineyards and the 2018 and 2019 growing seasons in the central valley of California. **A** Each of the colored counties represents a vineyard that was sampled. Within each county box (San Joaquin, Merced, and Madera) the scion/rootstock combinations are depicted, corresponding to the legend in the bottom left. Sugar content (measured in degrees Brix) was collected for vines across the **B** 2018 and **C** 2019 growing seasons; measurements and trend lines are colored by scion (upper panel) and site (lower panel). Grey shading represents collection windows for microbiome sampling

We sampled vines in three commercial vineyards located along a 177 km north–south transect running through Madera, Merced, and San Joaquin counties in central California (Fig. 1A). Each vineyard contained mature grafted vines (> 6 years old; Additional file 2: Table S1) composed of one of two scions (‘Cabernet Sauvignon’ or ‘Chardonnay’) and one of three rootstocks (‘Freedom’, ‘1103P’, or ‘Teleki 5C’; Fig. 1A; Table 1). Each vineyard has a unique vineyard design (Additional file 2: Table S1) and unique soil properties (Additional file 2: Table S2). The vineyards follow an environmental gradient with the most southern vineyard, Madera, being typically hotter (+ 2.6 °C) and dryer (− 7.4% relative humidity) than the most northern vineyard, San Joaquin (Additional file 1: Fig. S1). Conventional management practices such as mechanical leaf thinning and spray applications of pesticides and fungicides were employed at all vineyards.

**Table 1:** Parentage of rootstocks within this study (Source of parentage information: Foundation Plant services—University of California, Davis)

Rootstock	Parent 1	Parent 2
Teleki 5C	*berlandieri*†	*Riparia*
1103 Paulsen	*berlandieri*†	*Rupestris*
Freedom	*solonis*^b^ × *othello*^a^	*champinii*^c^

^†^*Vitis berlandieri* is also commonly known as *Vitis cineria* var. *helleri*

^a^*Vitis* × *othello* is of hybrid origin from a cross of *Vitis vinifera* × *Vitis labrusca/riparia*

^b^*Vitis solonis* is a synonym of *Vitis acerifolia*

^c^*Vitis* × *champinii* is of hybrid origin from a cross of *Vitis* *mustangensis* × *Vitis* *rupestris*)

One of the three vineyards (San Joaquin) contained all six scion/rootstock combinations (‘Cabernet Sauvignon’ grafted to ‘Freedom’, ‘1103P, and ‘Teleki 5C’; and ‘Chardonnay’ grafted to ‘Freedom’, ‘1103P, and ‘Teleki 5C’). Six collection blocks were sampled at the San Joaquin site (i.e*.*, sets of 24 vines, at least 10 vines from the edge, across 2–3 rows; Fig. 1A; Additional file 2: Table S1). In the other two vineyards, Madera and Merced, collection blocks for four of the six scion/rootstock combinations were sampled as not all scion/rootstock combinations were present at these vineyards (Fig. 1A; Additional file 2: Table S1). In Madera, blocks were sampled for ‘Cabernet Sauvignon’ grafted to ‘Teleki 5C’ and ‘Freedom’ and ‘Chardonnay’ grafted to ‘1103P’ and ‘Freedom’. In Merced, blocks for ‘Cabernet Sauvignon’ grafted to ‘1103P’ and ‘Freedom’ and ‘Chardonnay’ grafted to ‘Teleki 5C’ and ‘Freedom’ were established (Fig. 1A). From each collection block, three representative vines per scion/rootstock combination were selected. Different vines were sampled in each collection window to minimize disturbance of the vine and its microbiome from repeated collections. Care was taken to ensure that selected vines did not exhibit signs of pathogen infection. We staggered collection windows over the course of the season to capture vine development: in 2018 we made three collections three weeks apart from June 19th to August 2nd, 2018, and in 2019 we made two collections four weeks apart June 11th to July 31st, 2019 (Fig. 1B, C; Additional file 1: Fig. S2). Sampling periods coincided with multiple developmental stages for the vines, starting at early fruit formation to veraison and, for ‘Chardonnay’, early harvest.

Three compartments (roots, leaves, berries) were sampled from each vine. Care was taken to ensure each sample was collected in a sterile manner, tools were surface sterilized between samples using 95% ethanol and all samples were stored in sterile single use plastic bags. Roots were excavated to a depth of 20–30 cm using a sterilized shovel, and then several root segments were collected by hand with the aid of sterilized stainless-steel scissors. Roots segments were shaken to remove loosely attached soil prior to storage. Three to five leaves were clipped at the base (*i.e.*, removing the petiole and leaving only leaf blade) using sterilized stainless-steel scissors, leaves were approximately 8–12 cm in diameter and were collected at roughly the middle position along a shoot and at a height of 1.5 m on the vine. Berries were collected as an intact cluster, clipping the rachis of the cluster at the point of attachment with the shoot using sterilized stainless-steel pruning shears. We measured total soluble solids (sugar content) in °Brix using a hand-held refractometer (ATAGO) by selecting berries from a damage-free representative cluster on the same vine. Sugar content measurements were collected each time berry clusters were sampled as well as once per week at each vineyard to track berry development (Fig. 1B, C). An approximately 200–300 g soil sample was collected during the final sampling time point each year from between two vines, spaced approximately 1–2 m from either vine depending on vine spacing (Additional file 2: Table S1), for each collection block. Soil was collected at a similar depth to root collection, 20–30 cm, with a sterilized shovel, discarding the first 3–5 cm of topsoil, and passed through a sterile sieve (American Standard No. 16; 1 mm pore size). All samples were placed in a cooler with ice packs in the field prior to shipping on dry ice and storage at − 20 °C at the Danforth Plant Science Center (St. Louis, MO).

### Soil texture and elemental composition

Soil samples were split into two portions: one for molecular processing (see below) and one for texture and elemental composition analysis. Prior to texture and elemental composition analysis, soil was dried until a consistent weight was achieved, approximately one week at 70 °C. For texture analysis, 50 g of each soil sample was added to a screw-top jar along with 125 mL of sodium hexametaphosphate solution (Sigma-Aldrich; 0.065 M) and allowed to agitate overnight on a stir plate. The next day the contents of the jar were transferred to a 1L graduated cylinder and filled to 1L with deionized water. The cylinder was capped and agitated by inverting approximately 30 times for 1 min. A hydrometer (H-B Instrument Company, 152H) was placed in the cylinder, and the first reading was taken after 40 s. Readings were then taken periodically for the next six hours. Elemental composition analysis was conducted at the Agricultural Diagnostic Laboratory (Fayetteville, AR) following established protocols [69, 70] to determine concentrations (in ppm) of the following elements; B, Ca, Cu, Fe, K, Mg, Mn, Na, P, S, and Zn, along with pH.

### DNA extraction and 16S rRNA amplicon sequencing

Soil, root, leaf, and berry samples were processed using previously described methods [53]. DNA extractions were performed with the DNeasy Powersoil Pro Kit (Qiagen) following the manufacturers protocol with two modifications: we used 150 mg of plant tissue (non-surface sterilized; *i.e.*, containing both endophytes and epiphytes) per extraction, and we added a 10-min incubation at 70 °C prior to homogenization with a bead mill (Retsch, MM 400). DNA extracts were qualified on a DS-11 Spectrophotometer (DeNovix) and sent to the Environmental Sample Preparation and Sequencing Facility at Argonne National Laboratory for 16S rRNA amplicon sequencing. Amplicon sequencing and library preparation were conducted following Swift et al*.* (2021). Samples were split into two pools of 336 samples each for sequencing conducted on an Illumina MiSeq, with a 2 × 151 bp Pair-End kit. Samples that produced fewer than 20,000 reads after preliminary quality control and filtering were combined into a third pool that was sequenced on an additional flow cell.

### Amplicon processing and ASV filtering

Sequence processing was conducted using a similar workflow to Swift et al*.* [53]. Briefly, QIIME2 v2021.4 [71] was used to demultiplex samples according to barcode sequence. The DADA2 plugin [72] in QIIME2 was used to denoise, dereplicate, and filter chimeric sequences on each sequencing run individually for accurate error model generation. The resulting amplicon sequence variant (ASV) tables and catalogs of representative sequences for each sequence plate were merged. A Naive Bayes classifier pre-trained on the SILVA v.138 16S rRNA gene database [73, 74] was used for taxonomic classification. ASVs not assigned to a phylum were removed along with ASVs assigned to chloroplasts and mitochondria (2.2% and 1.9%, respectively), resulting in 45,332 ASVs. The Decontam v1.12.0 package [75] was used to remove contaminants using the prevalence-based detection method with a threshold of 0.5, removing contaminant ASVs more prevalent in the negative controls than real samples. Decontam identified 183 ASVs as contaminants. The data set was filtered to remove singletons by retaining only ASVs present in five or more samples and by removing samples with a read count less than 1,000.

### Statistical analysis

All analyses were conducted within the R environment v4.1.0 [76]. We first modeled the sugar content of the berries of the vines across the growing season. Using a linear model, we assessed effects from the experimental design (rootstock, scion, site, collection week, and their interactions) on sugar content (°Brix). The car package v3.0-11 [77] was used to assess the model under a type-3 ANOVA framework. For significant terms in this model, and all other models, we conducted post hoc comparisons of estimated marginal means using the emmeans package v.1.6.2.1 [78], utilizing a Tukey-correction for multiple comparisons.

For soil texture, hydrometer readings were processed using the envalysis package v0.5.1 [79] to obtain percentages of sand, silt, and clay. Independent linear models for sand, silt, and clay were fit with collection site and year as main effects. For elemental composition analysis, concentration more than five standard deviations from the mean were removed. A biplot was generated using the factoextra package v1.0.7 [80] to visualize clustering of soil samples by collection site along with the loadings of the principal component analysis (PCA). A linear model was fit to the first two principal components (PC) with collection site and year as main effects. Each of the linear models was assessed via a type-2 ANOVA framework.

ASV counts were normalized by applying a variance stabilizing transformation from the package DESeq2 [81] with a model containing all of the main effects (Rootstock + Scion + Compartment + Year + Site + Sugar Content). Alpha diversity statistics, Chao1 and Faith’s Phylogenetic distance [82], were calculated using vegan v2.5.1 [83] and picante v1.8.2 [84], respectively. Linear mixed models were fit via the lmerTest package v3.1.3 [85] to assess the effects of the experimental design on alpha diversity indices (response ~ Rootstock (R) + Scion (S) + Compartment (C) + Sugar Content (Su) + Year + Site + R × S + C × Su).

We used principal coordinate analysis (PCoA) with Bray–Curtis dissimilarity in order to visualize clustering of samples across experimental factors. PERMANOVA analyses were conducted using the function *Adonis* from the package vegan. For each plant compartment (berries, leaves, roots) a model with Bray–Curtis dissimilarity as the response was fit with all factors as marginal fixed effects, using 1,000 permutations per model. Using a linear model framework, we examined the abundance of each of the top ten bacterial phyla by relative abundance. A linear model was fit with rootstock, scion, brix, year, and site as fixed effects with each phylum as the response variable. The *P-*values from all tests, across phyla, were corrected for multiple testing using false discovery rate [86].

### Machine learning

For machine learning, categorical factors are preferred over continuous factors to allow for easier statistical interpretation. As such, we discretized berry sugar content values. To choose where to split sugar content values into groups, we plotted values and chose the natural break point in the values. Samples were given the labels pre-ripening (3–7°Bx; n = 357) and ripening (> 7°Bx; n = 237; Additional file 1: Fig. S3).We used ranger’s v0.13.1 [87] implementation of the random forest algorithm in the caret package v6.0.90 [88] to assess predictability of sample labels and identify ASVs that contribute to prediction accuracy. For training the random forest classifier, the dataset was randomly split into a training set (80%) and a testing set (20%). Optimal hyperparameters for each classifier were determined using a grid search over the number of trees (1–501; Additional file 1: Fig. S4), minimum node size (1, 5, 10), and number of features available at each node (10–100% of the ASVs). For each combination in the grid, performance of the classifier on out-of-bag samples was assessed with tenfold cross validation. Classifiers were then trained to predict each of the categorical factors (rootstock, scion, compartment, year, and site) along with all possible pairwise joint predictions. Tile plots were used to visualize output confusion matrix results. We determined relative importance of phyla in classification accuracy per factor, as well as ASVs that contributed considerably to classifier accuracy (i.e., high gini importance).

## Results

### Soil properties and soil microbiome showed site-specific differences

**Fig. 2:**
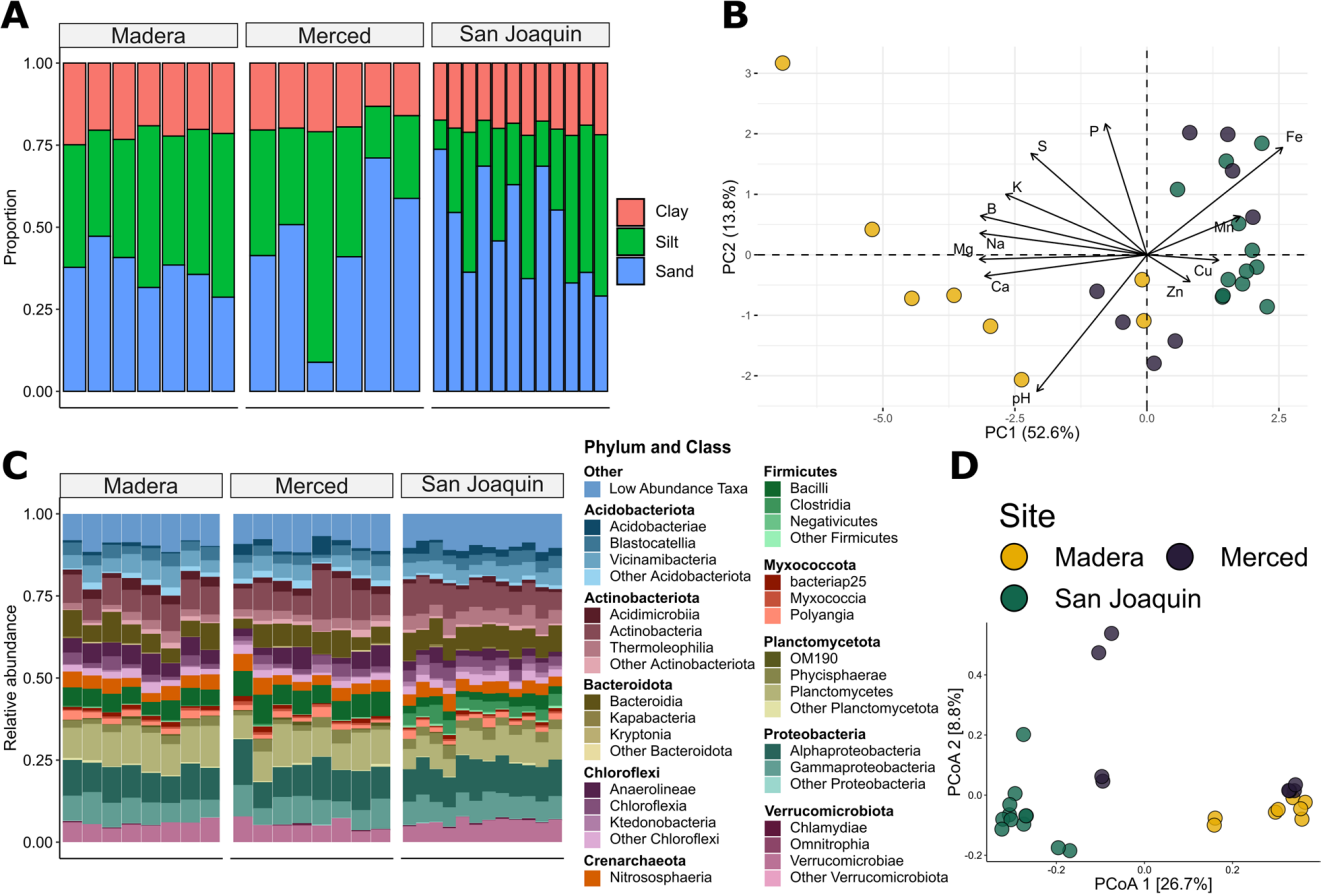
Soil properties and microbiome for the collection sites. **A** The soil texture measured in proportions of sand, silt, and clay and **B** a principal component analysis of soil elemental composition and pH, arrows represent loadings of elements and pH on principal components 1 and 2. **C** Taxonomic barplots depicting the relative abundance of the top ten phyla subdivided into the top three classes, phyla below the top ten are condensed into the “Low Abundance Taxa” category. **D** Principal coordinate analysis on Bray–Curtis dissimilarity for soil samples. For both panels B and D, the colors correspond to collection sites

Soil texture, elemental composition and the diversity and richness of the soil microbiome differed across collection sites and by year. Soil texture was modeled in proportions of sand, silt, and clay, and clay was significantly different between collection sites (Fig. 2A; Additional file 2: Table S4; *P* = 0.041). Post-hoc comparisons revealed a 3.3% increase in clay content in Madera as compared to Merced (Fig. 2A; *P* = 0.035). Sand and silt showed no significant differences across collection sites or years (Additional file 2: Table S4). The principal component analysis of the elemental composition data showed clustering of samples by collection site (Fig. 2B). PCs 1 and 2 collectively explained over 70% of the elemental variation (52.6% and 13.8% respectively), and each was with a linear model parameterized with collection site and year. Collection site showed a significant effect on the first PC (Additional file 2: Table S5;* P* < 0.001) but not the second PC (Additional file 2: Table S5), while collection year showed no significant effect on either PC. Post-hoc comparisons on the first PC model showed that the Madera site was differentiated from both Merced (*P* < 0.001) and San Joaquin (*P* < 0.001; Fig. 1A) whereas the Merced vs San Joaquin comparison was non-significant.

To assess the soil microbiome, we recovered 921,649 bacterial reads (n = 28; sample mean 32,916) across 4197 ASVs after quality filtering. The top ten most abundant bacterial phyla recovered from soil samples were shared between sites (Fig. 2C). Principal coordinate analysis of Bray–Curtis dissimilarity revealed clustering by site (R^2^ = 0.296); we observed tight groupings for Madera and San Joaquin samples, but observed more variance in Merced samples, with some clustering closer to Madera samples (Fig. 2D). PERMANOVA analysis further illustrated that differences in the composition of the soil microbiome were attributable to site (Additional file 2: Table S6; *P* < 0.001), while sample collection year was non-significant. Both bacterial richness and diversity were impacted by collection year (Additional file 2: Table S7; *P* = 0.028 and *P* = 0.039, respectively), with post-hoc comparisons showing that both richness and diversity were lower in 2018.

### Sugar content tracked vine development across the growing seasons

The sugar content of berries provided a standardized measure to assess development of the grapevines across season and site, as well as to characterize differences between scions ‘Cabernet Sauvignon’ and ‘Chardonnay’. The three-way interaction of collection site, scion, and collection week explained observed patterns in sugar content (Additional file 2: Table S8;* P* < 0.001). In the last collection week, ‘Cabernet Sauvignon’ was, on average, 4.957 ± 0.824°Brix lower in the northernmost collection site, San Joaquin, compared to °Brix observed in Madera and Merced (Fig. 1A). All post-hoc comparisons for ‘Cabernet Sauvignon’ were significant (*P* < 0.001) with the exception of ‘Cabernet Sauvignon’ in the last collection week of 2018 (San Joaquin vs Merced; *P* = 0.108). For ‘Chardonnay’, we found a similar pattern, with the last collection week showing the most divergence in °Brix between the sites, with San Joaquin on average 1.382 ± 0.813 lower than Madera and Merced (Fig. 1A). Post-hoc comparisons for ‘Chardonnay’ showed that only the comparison of San Joaquin to Merced in the last collection week of 2018 was significant (*P* = 0.033). Given that berry sugar content tracks vine development across sites, season, and scions, we elected to use this measure in place of collection week in subsequent linear models.

### Bacterial diversity and richness were impacted by compartment, year, and rootstock by scion interactions

We generated bacterial sequence data for root (n = 206), leaf (n = 204), and berry (n = 184) samples of grafted grapevines growing in three vineyards in the Central Valley of California. The resulting dataset comprised 7,981 ASVs and a total read count of 24,502,564 (sample mean 41,250). We observed a large overlap of ASVs from below-ground and above-ground compartments, with 36% of ASVs shared among roots and either berries, leaves, or both (Additional file 1: Fig. S5A). When including soil samples, we observed 7% of ASV shared among berry, leaf, root, and soil samples and the largest overlap of ASVs, 28%, to be between roots and soil (Additional file 1: Fig. S5B). Berries (1 ASV), leaves (14 ASVs), and soil (18 ASVs) had a low number of unique ASVs in contrast to roots (3105 ASVs; Additional file 1: Fig. S5B).

**Fig. 3:**
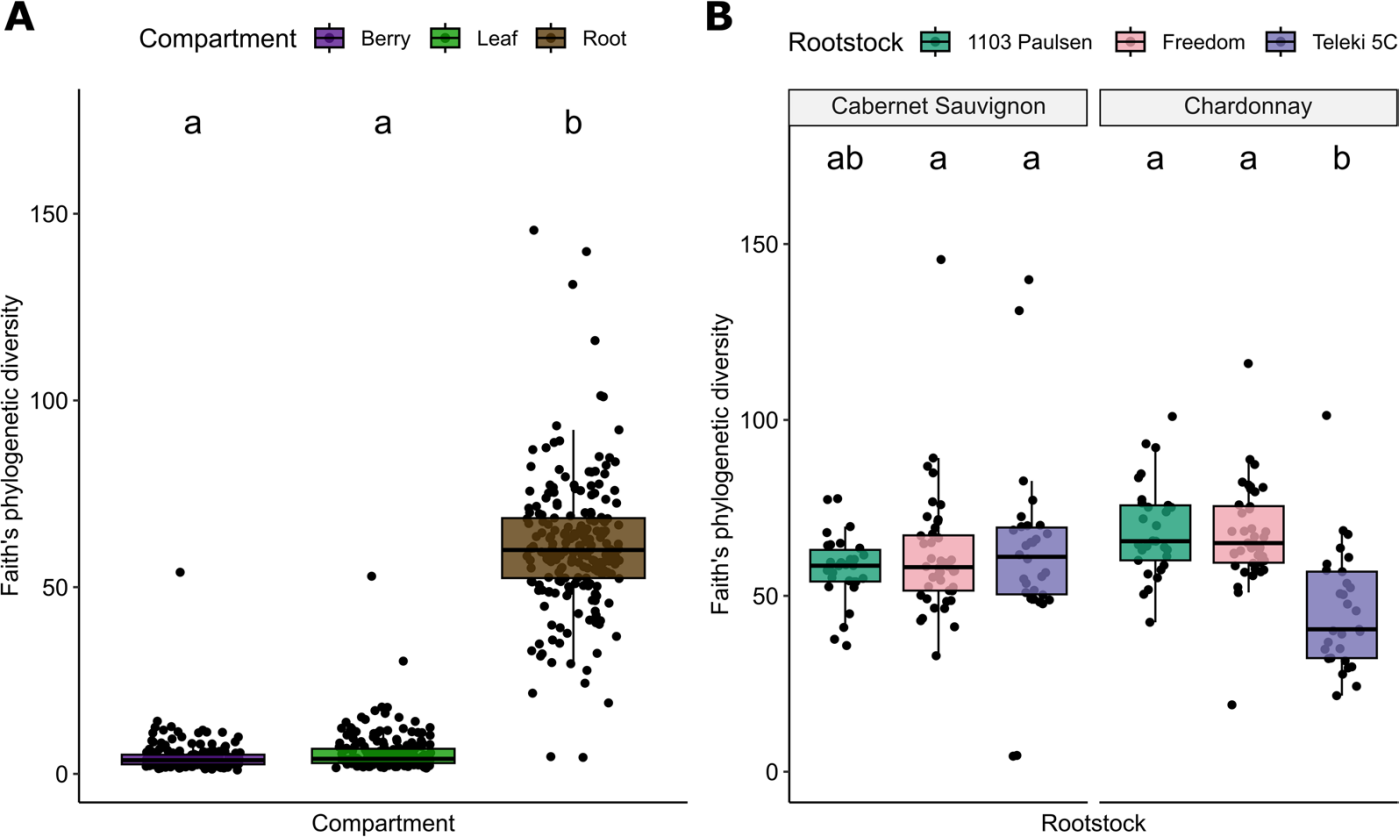
Faith’s phylogenetic diversity index was impacted by multiple factors of the experimental design. **A** Plant compartment showed the largest effect on diversity and **B** Root samples showed a rootstock by scion genotype effect on diversity. Boxplot hinges represent the 1st and 3rd quartiles with whiskers that represent 1.5 times the inter-quartile range; letters above box plots denote significant differences in means (TukeyHSD test)

Plant compartment had the largest impact on Faith’s phylogenetic diversity index and richness (Additional file 2: Tables S9 and S10; Fig. 3A). Root samples were significantly more diverse than both berries and leaves (Fig. 3A; *P* < 0.001) with mean values of 61.07, 4.63, and 5.71, respectively. Soil samples had a slightly lower level of diversity than roots with a mean value of 52.08. In addition to compartment, Faith’s phylogenetic diversity index also varied significantly across collection years (Additional file 2: Table S9; *P* < 0.001), with samples collected in 2018 having slightly higher diversity than samples collected in 2019. While site had a significant effect on Faith’s phylogenetic diversity index for plant compartments overall (Additional file 2: Table S9; *P* = 0.041), no post-hoc comparisons were significant among sites. The interaction between rootstock and scion genotypes also explained patterns of bacterial diversity (Additional file 2: Table S9; *P* < 0.001); post-hoc comparisons revealed that the rootstock ‘Teleki 5C’ grafted to the scion ‘Chardonnay’ had a lower mean bacterial diversity than other rootstocks (‘1103P’ vs ‘Teleki 5C’, *P* < 0.001 and ‘Freedom’ vs ‘Teleki 5C’, *P* < 0.001). This pattern was particular to the root samples (Fig. 3B) and partially explained by a specific vineyard block at the Merced vineyard, Mer-4 (Additional file 2: Tables S1 and S2) featuring ‘Chardonnay’ grafted to ‘Teleki 5C’. Root samples within this block had a mean Faith’s phylogenetic diversity index of 37.10 ± 5.15, whereas the same rootstock/scion combination at the San Joaquin vineyard was 54.05 ± 2.39. Due to the experimental design, we are not able to fully disentangle the effects of particular vineyard blocks from rootstock/scion combinations. Although, the soil pH of this block was the lowest of the study at 5.7 (overall mean = 7.6; Additional file 2: Table S2).

### Bacterial composition of the root varied by site and by rootstock genotype but was relatively stable over time

**Fig. 4:**
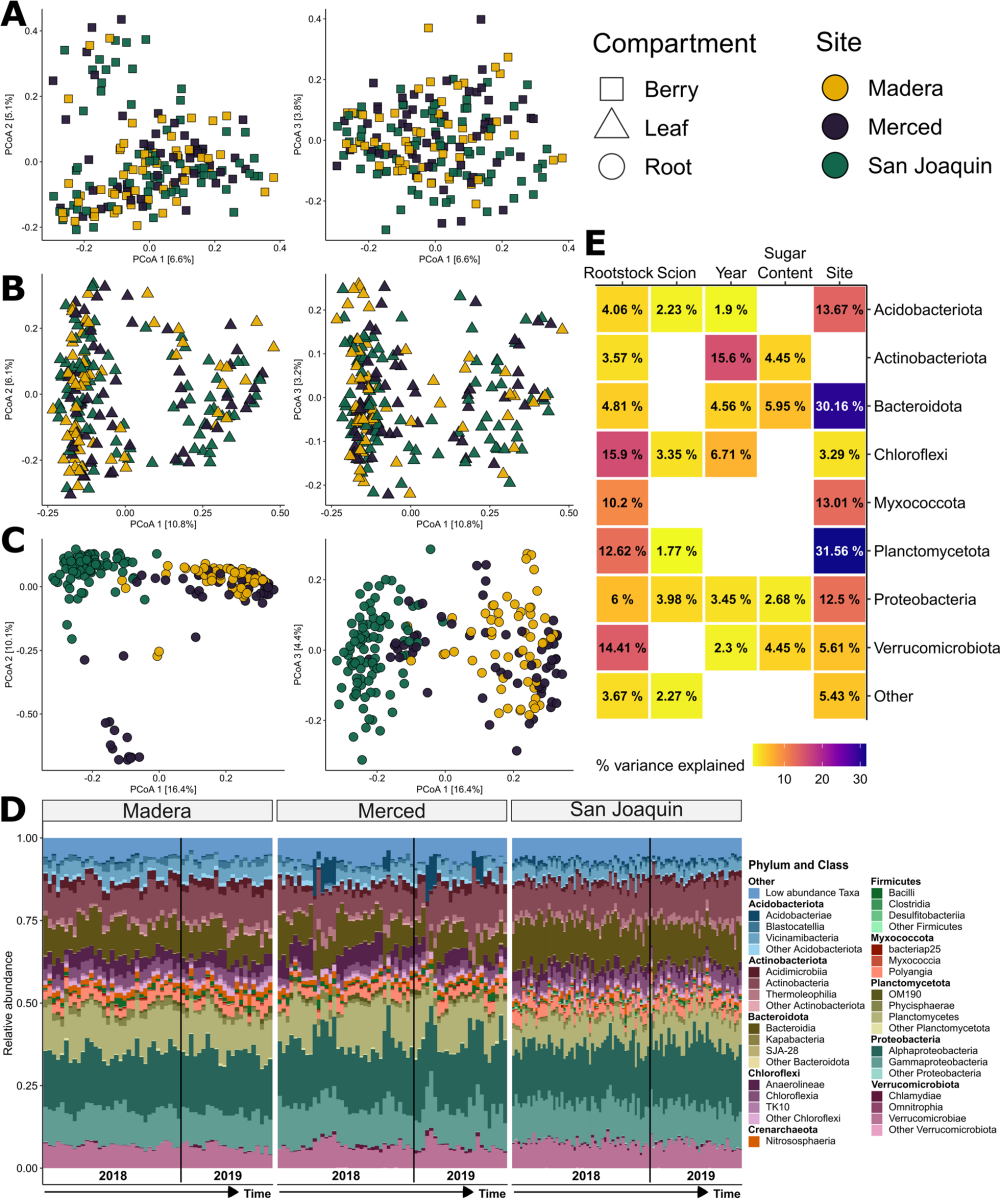
Principal coordinate analysis (PCoA) by sample compartment of Bray–Curtis dissimilarity. **A** Berry, **B** leaf and **C** root samples, only the root compartment displays clustering by collection site (right panels, Axis 1 *v.* 2; left panels, Axis 1 *v.* 3). For PCoA plots displaying compartments together and additional experimental factors, see Additional file 1: Figs. S6 and S7, respectively. Taxonomic barplots with the relative abundance of the top ten phyla subdivided into the top three classes for root samples, **D** delineated by collection site, each bar represents an individual sample and samples are ordered according to collection date with the black line denoting collection year. Phyla below the top ten are condensed into the “Low Abundance Taxa” category. **E** A tile plot summarizes the significant (*P*_FDR_ < 0.05) impact of the experimental factors on the relative abundance of the top ten phyla (Deinococcota and Firmicutes are omitted as no factors were significant)

Principal coordinate analysis on Bray–Curtis dissimilarity values showed clear clustering of the bacterial composition of samples by plant compartment on axis 1 and 2 (Additional file 1: Fig. S6A), explaining 17.5% and 4.6% of the variance, respectively. The third axis clustered root samples by the site of collection (Fig. 4C and Additional file 1: Fig. S6B), with San Joaquin separated from Madera and Merced. This pattern was unique to the root samples as berries and leaves were tightly clustered together on axis 3 (Fig. 4A, B). The bacterial composition of the root compartment samples was relatively stable over the course of the growing seasons but was variable within a site, particularly Merced, and across the sites (Fig. 4D; Table 2). Interestingly, for the Merced block (Mer-4) with the lower-than-average pH, we observed an enrichment of the bacterial class Acidobacteriae and depletion of the phylum Chloroflexi (e.g., classes Anaerolineae and Chloroflexia), which may relate to pH preferences for members these groups [89–91].

**Table 2:** Permutational multivariate analysis of variance (PERMANOVA) using Bray–Curtis dissimilarity fit separately for each compartment

Factor	Berry		Leaf		Root	
	**R**^**2**^	***P***	**R**^**2**^	***P***	**R**^**2**^	***P***
Rootstock	0.011	0.474	0.010	0.258	**0.046**	**0.001**
Scion	0.007	0.110	0.005	0.280	**0.018**	**0.001**
Sugar Content	**0.014**	**0.001**	**0.010**	**0.001**	**0.006**	**0.032**
Year	**0.015**	**0.001**	**0.034**	**0.001**	**0.014**	**0.001**
Site	0.012	0.175	0.012	0.062	**0.166**	**0.001**
Residual	0.943		0.928		0.713	

Bold values indicate significant results, *P* < 0.05

We examined the impact of experimental factors on the top ten phyla based off relative abundance for root samples (Fig. 4E). Collection site impacted eight of the ten phyla and had the strongest impact overall on Planctomycetota (variation explained [VE] = 31.56%; *P*_FDR_ < 0.001), Bacteroidota (VE = 30.16%; *P*_FDR_ < 0.001), Acidobacteriota (VE = 13.67%; *P*_FDR_ < 0.001), Myxococcota (VE = 13.1%; *P*_FDR_ < 0.001), and Proteobacteria (VE = 12.5%; *P*_FDR_ < 0.001; Fig. 4E). As expected, given the clustering in the PCoA (Fig. 4C), the post-hoc comparisons of the other sites with San Joaquin were nearly all significant. Only Myxococcota and Proteobacteria showed different patterns, with post-hoc comparisons showing significant differences for Merced instead when compared to other sites. Only Deinococcota and Firmicutes, which together account for an average of 6.53% of relative abundance in root samples, had no significant associations.

Next, we examined the influence of rootstock genotype and scion genotype on the relative abundance of the top ten phyla of the root compartment. Rootstock genotype was an important factor explaining the relative abundance of eight of the top ten phyla, particularly Chloroflexi (VE = 15.9%; *P*_FDR_ < 0.001), Verrucomicrobiota (VE = 14.41%; *P*_FDR_ < 0.001), Planctomycetota (VE = 12.62%; *P*_FDR_ < 0.001), and Myxococcota (VE = 10.2%; *P*_FDR_ < 0.001; Fig. 4D). Post-hoc comparisons showed that these differences were driven primarily by ‘Teleki 5C’, with higher relative abundances for Chloroflexi (mean = 1.9% increase), Planctomycetota (mean = 1.87% increase), and Myxococcota (mean = 0.57% increase) and lower relative abundance for Verrucomicrobiota (mean = 1.01% decrease). Compared to rootstock genotype, scion genotype had a smaller impact on relative abundance of the top ten phyla recorded in the roots. Only four of the ten phyla of the root compartment showed significant impacts for scion genotype. For Acidobacteria, Chloroflexi, Planctomycetota, and Proteobacteria, scion cultivar explained less than 5% of the variance (Fig. 4D). Post-hoc comparisons showed that ‘Cabernet Sauvignon’ had a larger relative abundance of Acidobacteria, Chloroflexi, and Planctomycetota but the difference for each phylum was < 1% between scion genotypes, while Proteobacteria had a smaller relative abundance (mean = 1.16% decrease).

Finally, we examined the influence of two aspects of time, collection year, and sugar content of the berries (an analog for vine development within a season) on the relative abundance of the top ten phyla of the root compartment (Fig. 4E). Collection year significantly impacted six of ten phyla. Collection year had a particularly strong impact on the relative abundance of Actinobacteria (*P*_*FDR*_ < 0.001) explaining 15.6% of the variance (Fig. 4E). The 2019 collection year showed enrichment of Actinobacteria in comparison to 2018 (mean = 2.73% increase). In comparison, sugar content was among the least impactful factors, with only four of the ten phyla showing significant impacts of this factor. Sugar content explained < 6% of the variance for Actinobacteriota, Bacteroidota, Proteobacteria, and Verrucomicrobiota (Fig. 4E). Additional root compartment modeling across taxonomic levels can be found in Additional file 2: Table S11.

### Bacterial composition of berries and leaves showed less predictable patterns across sites

**Fig. 5:**
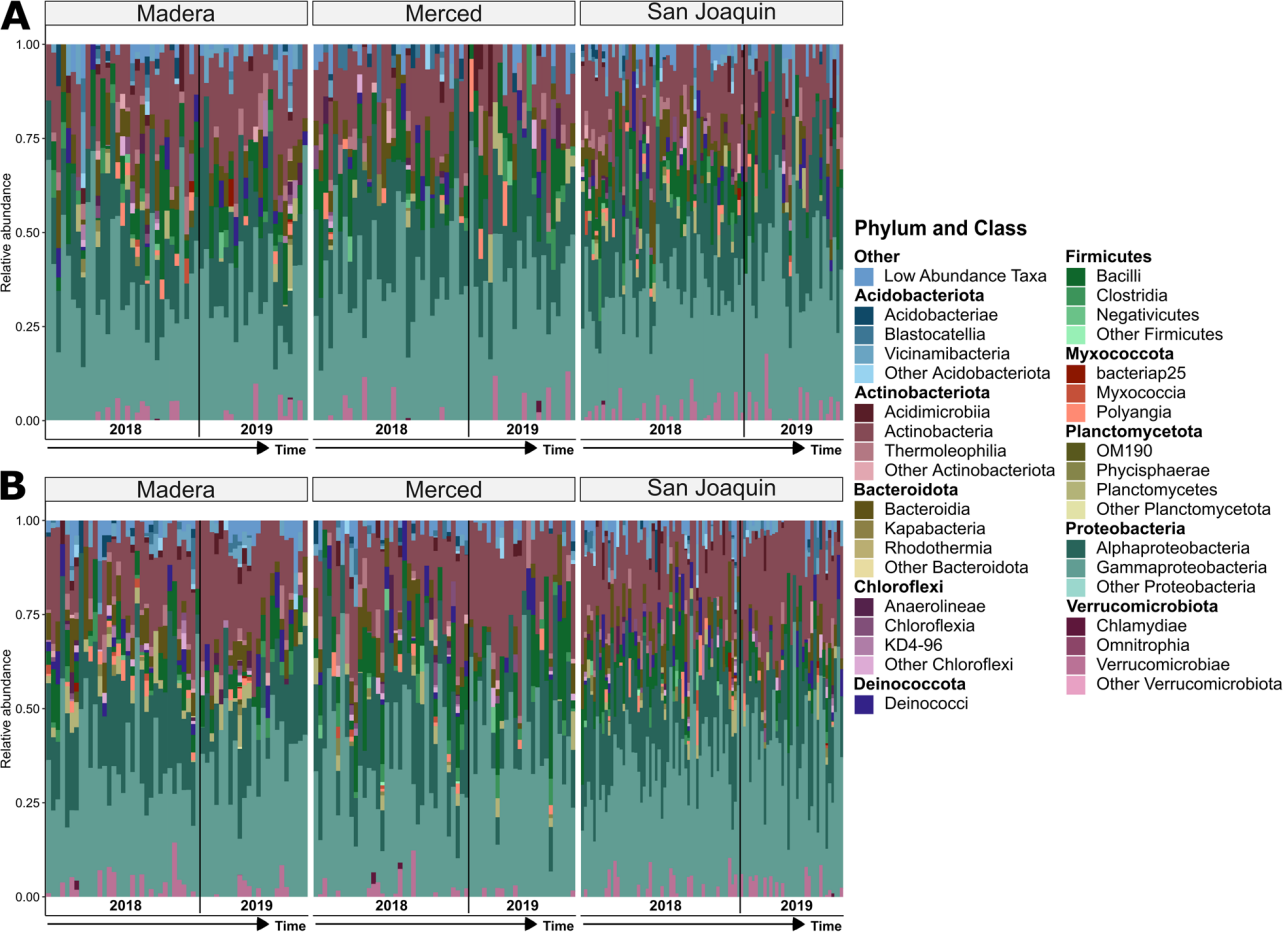
Taxonomic barplots with the relative abundance of the top ten phyla subdivided into the top three classes for **A** berry and **B** leaf samples delineated by collection site. Each bar represents an individual sample and samples are ordered according to collection date with the black line denoting collection year. All phyla below the top ten are condensed into the “Low Abundance Taxa” category

Principal coordinate analysis showed clear clustering of bacterial communities by plant compartments (Additional file 1: Fig. S6C and D). Bacterial composition of the root showed a clear signature of site; however, we did not observe the same clustering in relation to site for berry and leaf samples (Fig. 4A, B). For berries and leaves, PERMANOVA analysis showed a small effect of sugar content (Berries: R^2^ = 0.014, *P* = 0.001; Leaves R^2^ = 0.010, *P* = 0.001) and collection year on Bray–Curtis dissimilarity (Berries: R^2^ = 0.015, *P* = 0.001; Leaves R^2^ = 0.034, *P* = 0.001; Table 2). Taxonomic barplots of the top ten phyla for berry and leaf compartments showed fluctuations in relative abundance but were non-specific to collection sites (Fig. 5). Using the same linear model framework as above, we found that, for berries, only a single phylum, Bacteroidota showed a significant effect of collection year (VE = 4.12%: *P*_FDR_ = 0.029). Post-hoc comparisons revealed that berries in 2018 had on average 2% greater abundance of Bacteroidota. For leaves, only two phyla were impacted by the experimental factors. First, Firmicutes showed patterning by collection site (VE = 6.00%; *P*_FDR_ = 0.010), post-hoc comparisons of Firmicutes showed that the Merced site had, on average, 4.29% increased relative abundance compared to the other two sites (Fig. 1A) for leaf samples. Of note, Firmicutes did not show any significant impact of the experimental design for root samples (Fig. 4E). Second, Actinobacteriota showed patterning by the collection year (VE = 7.02%; *P*_FDR_ < 0.001), post-hoc comparisons of Actinobacteria showed a 5.42% decrease in relative abundance for 2018 versus 2019. Additional leaf and berry compartment modeling across taxonomic levels can be found in Additional file 2: Tables S12 and S13, respectively.

### Machine learning accurately predicts the root compartment but not berries or leaves

We used machine learning to identify the factors that were predictable across the experimental design (Additional file 1: Fig. S8) and the ASVs that aided the accuracy of those predictions (Additional file 1: Figs. S9 and S10). Overall, model accuracy was 68% (Additional file 1: Fig. S8A); however, the classifier was nearly perfect when predicting root samples (F1 = 0.986; Additional file 2: Table S14) and less accurate when predicting leaf (F1 = 0.525) and berry (F1 = 0.565). For rootstock genotype, model accuracy was 50% (Additional file 1: Fig. S8B) and ‘Freedom’ was the most predictable genotype (F1 = 0.623; Additional file 2: Table S14) while ‘1103 Paulsen’ and ‘Teleki 5C’ were considerably lower (F1 = 0.431 and 0.204, respectively). Model accuracy for the collection site was 59% (Additional file 1: Fig. S8C), the most predictable site was San Joaquin (F1 = 0.672), followed by Merced (F1 = 0.559) and then Madera (F1 = 0.431; Additional file 2: Table S14). For collection year model accuracy was 83% (Additional file 1: Fig. S6D) and an F1 score of 0.882 (Additional file 2: Table S15). For scion genotype, model accuracy was 61% (Additional file 1: Fig. S8E) and with an F1 score of 0.623 (Additional file 2: Table S12). Finally, for sugar content, model wide accuracy was 71% (Additional file 1: Fig. S8F) and an F1 score of 0.795 (positive class was pre-ripening; Additional file 2: Table S15).

When examining the phyla that were the most important in the classifier’s predictions, we found that across all factors many of the phyla have similar relative importance to their respective classifier (Additional file 1: Fig. S9). Proteobacteria was the most important phylum making between 39.7 and 54.6% of the relative importance to the classifier and Actinobacteria was the second most important phylum with between 14 and 24.9% of the relative importance to the classifier (Additional file 1: Fig. S9). Crenarchaeota was only important to the classification of plant compartments and collection sites with all ASVs showing affinity to the root compartment and some showing site-specificity. ASVs of this phylum (Additional file 1: Fig. S10), were annotated to the family Nitrososphaeraceae of which isolated members are known to oxidize ammonia and have been recovered previously from soils [92, 93].

## Discussion

The goal of this study was to investigate factors influencing bacterial communities of grapevine roots, leaves, and berries, including rootstock genotype, scion genotype, and vineyard site, within growing seasons and over multiple years. We observed differences among vineyard sites in soil texture, elemental composition, and bacterial communities. We detected differences in bacterial composition of grapevine root compartments across sites; however, site-specific differences were less pronounced in the microbiota of the berries and leaves. Both rootstock and scion genotype impacted composition and diversity of vine microbiota. Using Brix (berry sugar content) as a proxy for development, we observed only minor associations between developmental stage and bacterial community composition of the berries and leaves. This suggests that berry and leaf bacterial communities undergo largely stochastic changes in community composition across the season.

### Soils and the soil microbiome differ across sites

In the cultivation of wine grapes, the term “terroir” describes regional environmental factors, including soil properties, geography, and climate, that influence characteristics of wine [94–96]. These same factors influence the soil microbiome, a primary reservoir of microorganisms that colonize and ultimately become associated with plants [3, 14, 16, 97, 98]. Thus, many studies have uncovered a role for microorganisms in shaping the terroir of wine, often called the microbial terroir [46, 99, 100]. Previous research has shown that across regional scales, grapevine musts (crushed berry clusters) and wines exhibit site specific microbiota [46, 101–103], which are associated with the metabolomic composition of the wine [49, 104].

In this study, we demonstrated that vineyard soils within the Central Valley of California varied in elemental composition and soil texture (Fig. 2B, D). Further, differences in soil microbial communities were apparent across vineyards, an observation consistent with previous work in other viticultural regions [16, 45, 104, 105]. For instance, a recent global survey of vineyard soils across five continents [44], revealed prokaryotic communities bear a signature of spatial distance, with dissimilarity strongly positively correlated with geographic scales (regional to continental). Here, we found similar patterns at an intra-regional scale (~ 177 km apart between farthest vineyards) in the Central Valley of California. This work contributes to a growing body of literature documenting site-specific differences in biotic and abiotic properties of vineyard soils.

### Vineyard site influences bacterial composition of the grapevine root, but not berries or leaves

Within grapevines, bacterial composition bears a strong signature of compartment with roots, leaves, and berries exhibiting distinct microbiota [16, 53, 106]. Data presented here confirm that compartment specific dynamics primarily dictate the grapevine microbiota, an observation consistent with previous studies across plant species [4, 5]. Given differences in bacterial composition of different grapevine compartments, we might expect that compartment microbiota shift in unique ways in response to different geographic locations. For example, Zarraonaindia et al*.* [16] found that bacterial communities of the soil differed across several vineyards in the Northeastern United States, but those differences were not reflected in plant compartments. Conversely, a study conducted across the California coastal growing regions showed regional patterning for grapevine musts (crushed berries) for both bacterial and fungal communities [46]. The amount of variation explained by region and vineyard for bacteria was generally lower than for fungi, indicating that kingdoms exhibit different diversity patterns across local and regional environmental conditions [44].

Data presented here indicate that the bacterial community of the root was strongly influenced by vineyard site (Table 2; Fig. 3B, D), whereas bacterial communities of leaves and berries did not show site-specific patterning (Table 2; Fig. 5). Differences in how bacterial composition of specific compartments respond to vineyard location may reflect various biological and/or environmental factors. From a biological perspective, leaves and berries are short-lived compartments, developing anew annually, while the root system remains intact for a longer period of time. The senescence of leaves or harvest of berries may purge many bacterial community members established in a given season, restarting community succession and leading to more stochastic assembly patterns across years, while root communities remain intact. In addition, the root system is the only compartment assessed here that is in direct, sustained contact with the soil. Thus, it makes sense that the bacterial community of the root would more closely reflect the soil microbiome than the bacterial communities of the above-ground compartments of the vine, which were not in direct contact with the soil. While it has been shown that microorganisms can be transmitted from the soil to above-ground compartments via the roots [14, 107]. It is possible that filtering occurs, across the graft junction, by the scion genotype, as well as through selective pressure in the leaf and berry compartments, which may serve to obscure site-specific patterning of microbial communities from the roots to the shoot system.

Beyond the biology of the soil-root connections, there may be other viticultural and environmental factors that dictate compositional patterns in bacterial communities of above-ground vs. below-ground compartments [21, 108, 109]. Above-ground compartments were routinely spray treated to control pest and pathogen levels, as is common in conventionally managed vineyards, these treatments may alter the native microbial communities of leaves and berries [110, 111]. This disturbance in microbial communities, particularly epiphytic members, is likely to play a role in dictating compositional patterns in bacterial communities of leaves and berries. On the environmental side, rainfall serves as a dispersal mechanism for microorganisms, either through deposition from the atmosphere via precipitation [21, 112–114] or through water splashes containing soil [108, 115, 116]. Rainfall events catalyzed community succession in canola (*Brassica napus* L.) leaf microbiota [117]. In both 2018 and 2019, vineyards sampled for this study did not experience any precipitation during the sampling window and all hydration for the vine was supplied by drip irrigation. Consequently, it is unlikely that microbes would be dispersed from adjacent soil to aerial parts of the plant (leaves and berries) via water splashes at these vineyards during our sampling window.

A second possible environmental factor underlying our observation that root microbiota bear a signature of the geographic location, but leaves and berries do not, is the influence of the wind. Wind dispersal of soil microbiomes as observed in other plant systems [20, 118, 119] is a more likely factor in the Central Valley of California, as the majority of vines sampled in this study were covered in soil. A recent study found that airborne fungal microbiota (collected as settled dust) across the Central Valley of California were distinct from coinciding soil fungal microbiota, exhibiting remarkable similarity even from sites over 150 km apart [120]. Similarly, leaf and berry microbiota described here, despite variance in soil and root microbiota, are largely homogeneous, possibly due to airborne dispersal and deposition of dust/soil particles and the absence of physical disturbance by rain and water splashes.

### The influence of rootstock and scion genotype

Beyond the influence of the vineyard environment, there is evidence to suggest that both the rootstock and scion genotype play a role in shaping the bacterial communities in vine compartments. We detected an effect of both rootstock and scion genotype on bacterial composition of the root (Fig. 4; Table 2), as well as the bacterial diversity of the vine as a whole (Additional file 2: Tables S9 and S10). Interestingly, while the bacterial composition of root samples showed a signal of both rootstock and scion genotype, berry and leaf bacterial composition showed no significant patterning by either host genotype (Table 2). This is consistent with a recent study, where rootstock by scion interactions were associated with changes in the diversity and composition of root bacterial communities in grapevine [63] and previous studies examining rootstock-specific rhizospheres of multiple rootstock genotypes [59–61]. The phenotype most likely to drive host genotypic differences in vine bacterial composition is the root exudate profile, consisting of sugars, organic acids, and amino acids, among other compounds [7]. In *Arabidopsis thaliana*, root exudate variation was linked to the genomic variation among *A. thaliana* accessions [6] and has been associated with distinct rhizosphere bacterial communities [8].

Rootstocks studied here, and employed in most commercial vineyards, are of complex genetic backgrounds with two or more genetically distinct species serving as parents (Table 1; Riaz et al*.* [121]). Thus, there is likely substantial variation between the rootstock genotypes that may influence root exudate profiles, root architecture, and subsequently, the associated root microbiota. Relative to the rootstock genotype, the genotype of the grafted scion had a smaller overall influence on the composition of the root microbiome. This could be explained by proximity, with the rootstock genotype indirect contact with soil microorganisms. Further, compared to rootstock genotypes, the scion genotypes are more genetically similar to one another because both are cultivars of a single domesticated species, *Vitis vinifera*. Despite this, we found evidence for rootstock by scion interactions indicating both rootstock and scion genotypes contribute to the formation of the root microbiome. Experiments utilizing reciprocal rootstock/scion combinations can further serve to quantify the contribution of both the rootstock and scion in shaping the grapevine microbiome.

A limitation to our sample collection strategy is that we did not surface sterilize plant tissues, and as a result our plant samples contained a mixture of both endophytic and epiphytic bacteria. The assembly of leaf endophytic and epiphytic bacterial communities are governed to different degrees by both biotic and abiotic processes, with endophytic communities driven primarily by host plant identity whereas epiphytic communities are more influenced by site characteristics and dispersal patterns [122–125]. Similar findings have been recovered for grapevine berry and leaf epiphytic communities, with notable differences observed in the effect of host genotype (grapevine cultivar) based on the analysis scale, stronger within a viticultural zone and weaker at the larger national scale [126]. By sampling both endophytic and epiphytic bacterial communities together we limited our ability to differentiate factors shaping these two components of the grapevine microbiome, and only detected significant effects of factors that may act upon both communities simultaneously (*e.g.,* year and sugar content). Future studies designed to separate endophytic and epiphytic bacterial communities will provide further insights into biotic and abiotic contributions in shaping the grapevine microbiome.

### Bacterial diversity is stable over the growing season and may be decoupled from patterns of fungal diversity

By sampling across the growing season over multiple years, our experimental design allowed us to investigate the progression of bacterial communities of multiple compartments of the vine. We used berry sugar content to track vine development across the growing season and determined that the bacterial diversity of grapevine compartments generally do not shift based on vine developmental stage (Additional file 2: Tables S9 and S10). Despite this, we observed compartment specific temporal shifts in bacterial composition, however, sugar content and year explained only a small proportion of the variance (Table 2). Given that our experimental factors only captured a small amount of the variation in bacterial composition of both berries and leaves, this may indicate that community composition of these aerial compartments reflect stochastic processes and/or are influenced by environmental factors that were not measured over the course of this study (e.g., wind dispersal).

Data presented here focus on the bacterial communities of the grapevines. It is important to note that results for bacteria presented here and elsewhere [127], contrast with some patterns of fungal communities, where plant development has been shown to be a stronger predictor of shoot system community composition [32]. Other studies examining the microbiota of the berry epidermis reported associations between development stages and fungal [128, 129] and bacterial communities [130]. Given the sample size here (berries n = 184; leaves n = 204), we should be able to detect even subtle changes in community diversity and composition across vine development. However, it is possible that the sampling window may have been too narrow. Including samples from the earliest developmental stages (leaf flush and fruit set) to latest stages (senescence and harvest), may have resulted in a stronger signature of development on the microbiota.

## Conclusions

Uncovering factors shaping plant-associated microbiomes has implications for understanding how plants establish and respond to their environments, in both natural and agricultural settings. Work described here demonstrates site-specific biogeographical patterns in bacterial communities in the soil, as well as corresponding patterns in the root compartment of grafted grapevines. However, bacterial communities of berries and leaves reflected patterning by development and collection year, not site. This result illustrates that the microbial community composition of different plant compartments are impacted uniquely by the local conditions of a particular geographic location. In addition, we show that grapevine rootstock genotype more strongly influences the bacterial communities of the compartments than the scion genotype, providing further resolution into the processes underlying microbial terroir in grafted grapevines. Future studies should leverage experimental designs that allow simultaneous sampling of multiple plant compartments across both space and time along with metagenomic techniques to probe the functional significance of differences between microbiomes.

### Supplementary Information

**Additional file 1**. **Figure S1** Environmental parameters for each vineyard. **Figure S2** Collection timeline for 2018 and 2019. **Figure S3** Discretization of sugar content values. **Figure S4** Out-of-bag error estimates for random forest models across values for the number of trees. **Figure S5** Venn diagrams depicting the overlap of ASVs between compartments and soil. **Figure S6** Principal coordinate analysis of Bray-Curtis dissimilarity for all compartments. **Figure S7** Principal coordinate analysis of Bray-Curtis dissimilarity each experimental factor across compartments. **Figure S8** Confusion matrices for each experimental factor. **Figure S9** Relative importance of phyla to random forest classifiers for each experimental factor. **Figure S10** Individual ASVs contributing to the accuracy of random forest classifiers for each experimental factor.

**Additional file 2**. **Table S1** Characteristics of the vineyard blocks used within the study. **Table S2** Soil elemental composition of the vineyard blocks used within the study. Values are reported in mg/kg (ppm) with the exception of pH which is reported in the standard scale. **Table S3** Optimal hyperparameters for training machine learning models. **Table S4** Linear model results for soil texture. Type-2 ANOVA table. **Table S5** Linear model results for soil elemental composition principal components (PC). Type-2 ANOVA table. **Table S6** Linear model results for Bray-Curtis Dissimilarity for soil samples. Type-2 ANOVA table. **Table S7** Linear model results for alpha diversity statistics for soil samples, Chao1 index and Faith’s phylogenetic diversity. Type-2 ANOVA table. **Table S8** Linear model results for sugar content of the berries. Type-3 ANOVA table. **Table S9** Linear mixed model results for Faith’s Diversity index. Type-3 ANOVA table. **Table S10** Linear mixed model results for Chao1 index. Type-3 ANOVA table. **Table S11** Linear model results for root compartment taxa across taxonomic levels for each experimental factor. **Table S12** Linear model results for leaf compartment taxa across taxonomic levels for each experimental factor. **Table S13** Linear model results for berry compartment taxa across taxonomic levels for each experimental factor. **Table S14** Output statistics for three-class machine learning models predicting rootstock genotype, collection site, and plant compartment. **Table S15** Output statistics for binary class machine learning models predicting scion genotype and collection year. In these models Cabernet Sauvignon and 2018 represented the positive class.

## Data Availability

All raw sequencing data is available on NCBI SRA under BioProject ID PRJNA849941 and SRA accessions SRR19735936-SRR19736586. All code to reproduce the analysis and figures is available on GitHub at https://github.com/Kenizzer/California_Transect_Microbiome.
