# Supplementary material for: Grapevine bacterial communities display compartment-specific dynamics over space and time within the Central Valley of California
Source: Environ Microbiome. 2023 Nov 23;18:84. doi: 10.1186/s40793-023-00539-0 (PMC10668525; doi:10.1186/s40793-023-00539-0)
Supplement: Supplementary file 1 — Additional file 1. Figure S1 Environmental parameters for each vineyard. Figure S2 Collection timeline for 2018 and 2019. Figure S3 Discretization of sugar content values. Figure S4 Out-of-bag error estimates for random forest models across values for the number of trees. Figure S5 Venn diagrams depicting the overlap of ASVs between compartments and soil. Figure S6 Principal coordinate analysis of Bray-Curtis dissimilarity for all compartments. Figure S7 Principal coordinate analysis of Bray-Curtis dissimilarity each experimental factor across compartments. Figure S8 Confusion matrices for each experimental factor. Figure S9 Relative importance of phyla to random forest classifiers for each experimental factor. Figure S10 Individual ASVs contributing to the accuracy of random forest classifiers for each experimental factor. [file 40793_2023_539_MOESM1_ESM.docx]

**Grapevine bacterial communities display compartment-specific dynamics over space and time within the Central Valley of California**

Joel F. Swift^1,2,4*^, Zoë Migicovsky^3,5^, Grace E. Trello^1^, and Allison J. Miller^1,2*^

^1^ Department of Biology, Saint Louis University, 3507 Laclede Avenue, St. Louis, MO, 63103, USA

^2^ Donald Danforth Plant Science Center, 975 North Warson Road, St. Louis, MO, 63132, USA

^3^ Department of Plant, Food and Environmental Sciences, Faculty of Agriculture, Dalhousie University, Truro, NS, B2N 5E3, Canada

^4^ Present address: Kansas Biological Survey & Center for Ecological Research, University of Kansas, Lawrence, KS 66045, USA

^5^ Present address: Department of Biology, Acadia University, Wolfville, Nova Scotia, B4P 2R6, Canada

**^*^Authors for correspondence:**

Joel F. Swift

joel.swift@ku.edu

Allison J. Miller

amiller@danforthcenter.org

# Supplementary Figures

**Figure S1.** Environmental parameters for each vineyard and year were extracted from the TerraClimate database [1], selecting the nearest 4x4 km grid cell to each vineyard. Daily environmental parameters **A)** Mean Temperature (Site *P* < 0.001, ANOVA), **B)** Evapotranspiration (Site *P* < 0.001, ANOVA), **C)** Downward Shortwave Radiation (Site *P* = 0.024, ANOVA), and **D)** Mean Relative Humidity (Site *P* < 0.001, ANOVA). From left to right, data are plotted for 2018 and 2019, respectively. Grey shading represents collection windows for microbiome sampling.


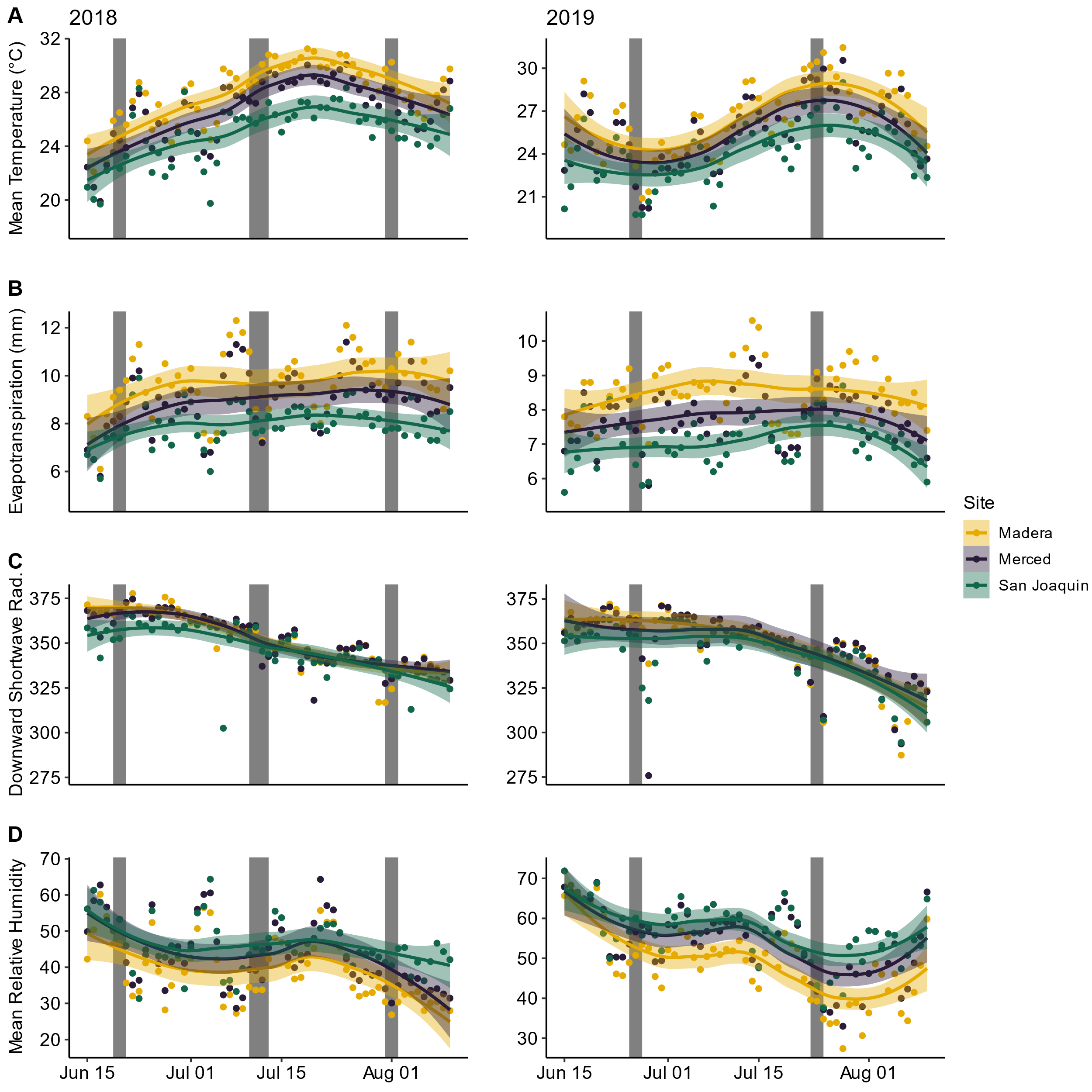


**Figure S2.** Collections were staggered across the growing season to capture the development of the vines. Photos display the progress of the vines for collections in 2018.

**
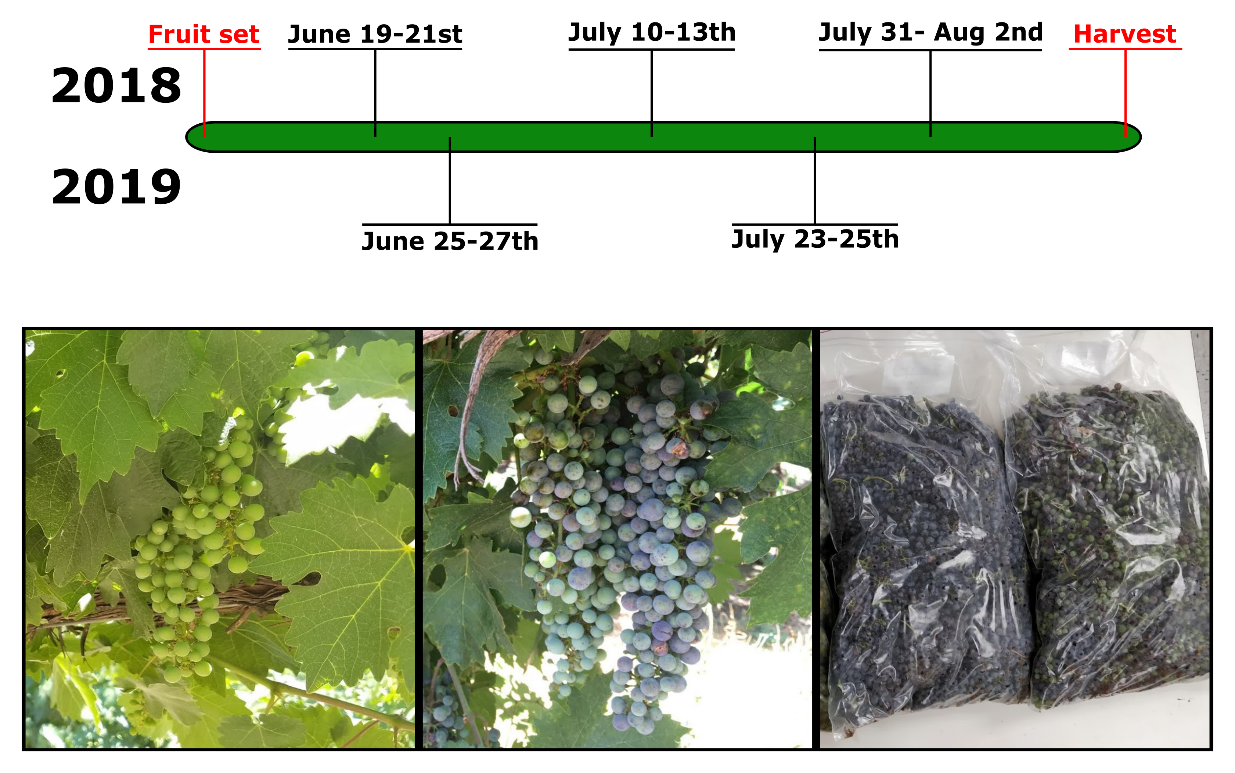
**

**Figure S3.** Discretization of sugar content values. The horizontal dashed line denotes where sugar content values (measured in °Bx) for samples were split in order to discretize for statistical analysis, values 3-7°Bx were labeled pre-ripening and values >7°Bx were labeled ripening. Points are colored according to the collection site.


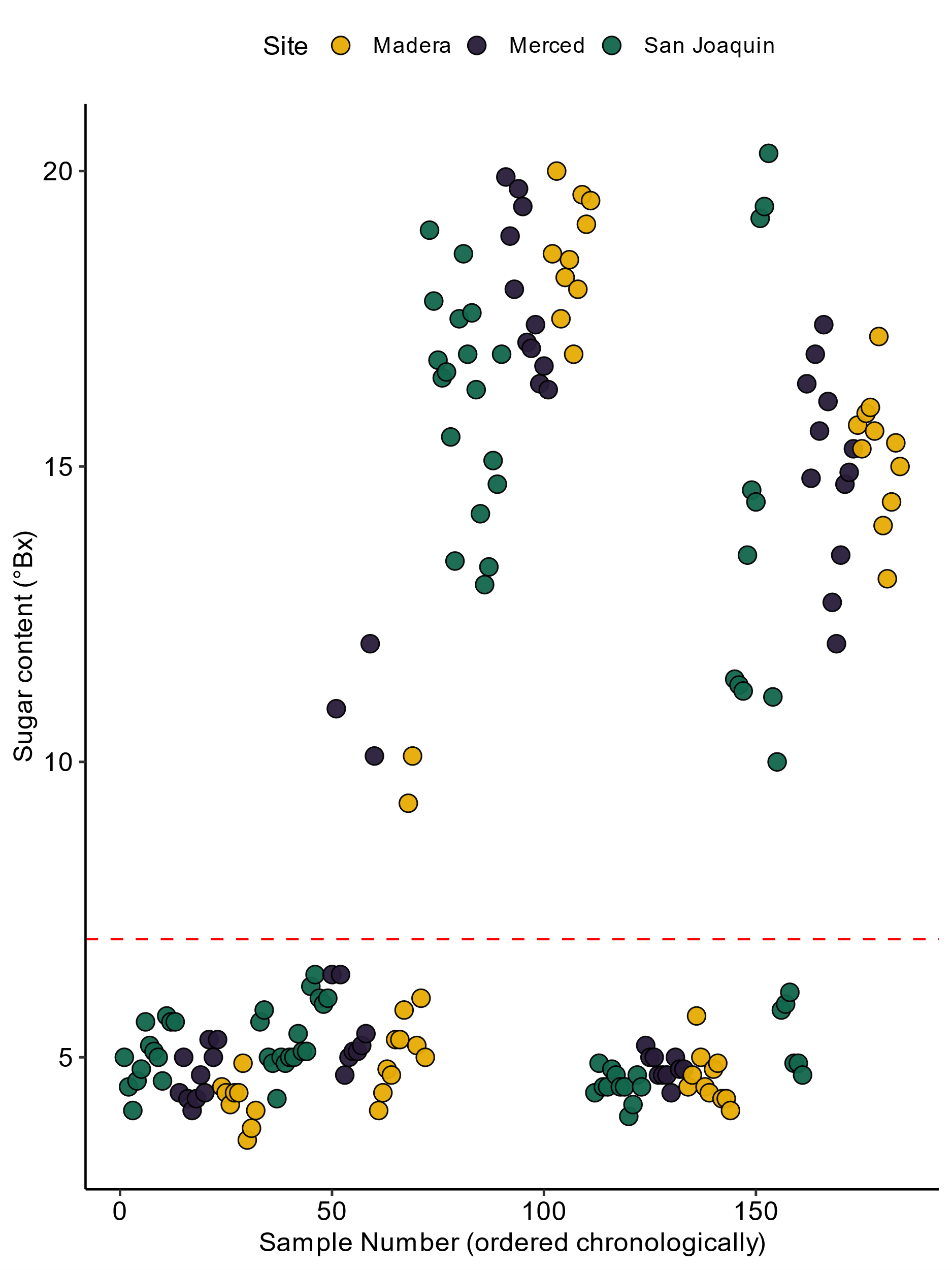


**Figure S4.** Out-of-bag error estimates for random forest models, across values for the number of trees, attempting to predict collection site, rootstock genotype, plant compartment, collection year, scion genotype, and sugar content. Dashed lines represent the minimum error estimate returned per model.


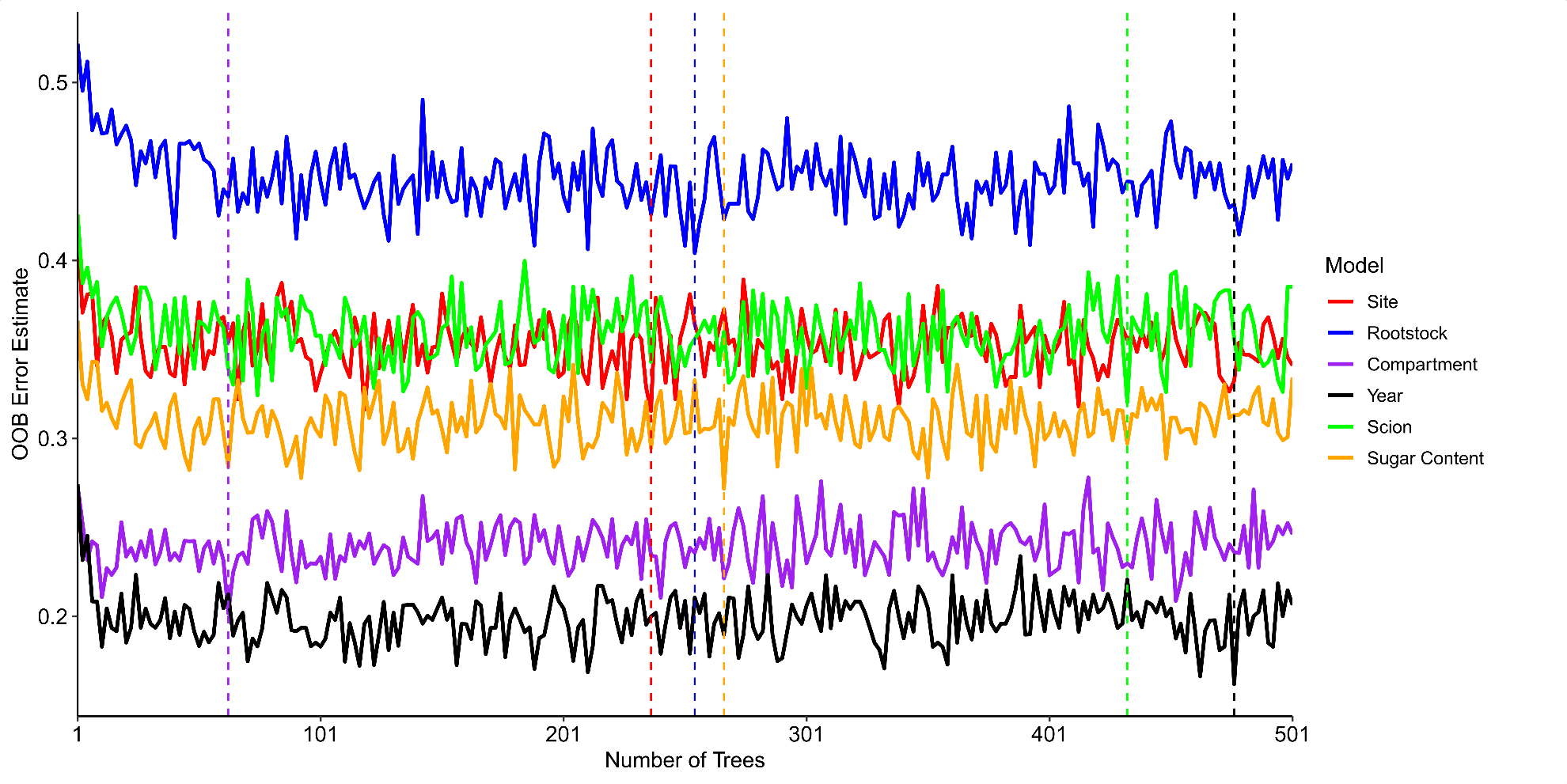


**Figure S5.** Venn diagram depicting the overlap of ASVs between compartments and soil. **A)** ASV overlap based on restricting ASVs to only those present in five or more samples when including grapevine compartments only (N = 594, total ASVs retained = 7981), **B)** ASV overlap based on restricting ASVs to only those present in five or more samples when including both grapevine compartments and soil samples (N = 622, total ASVs retained = 8838).


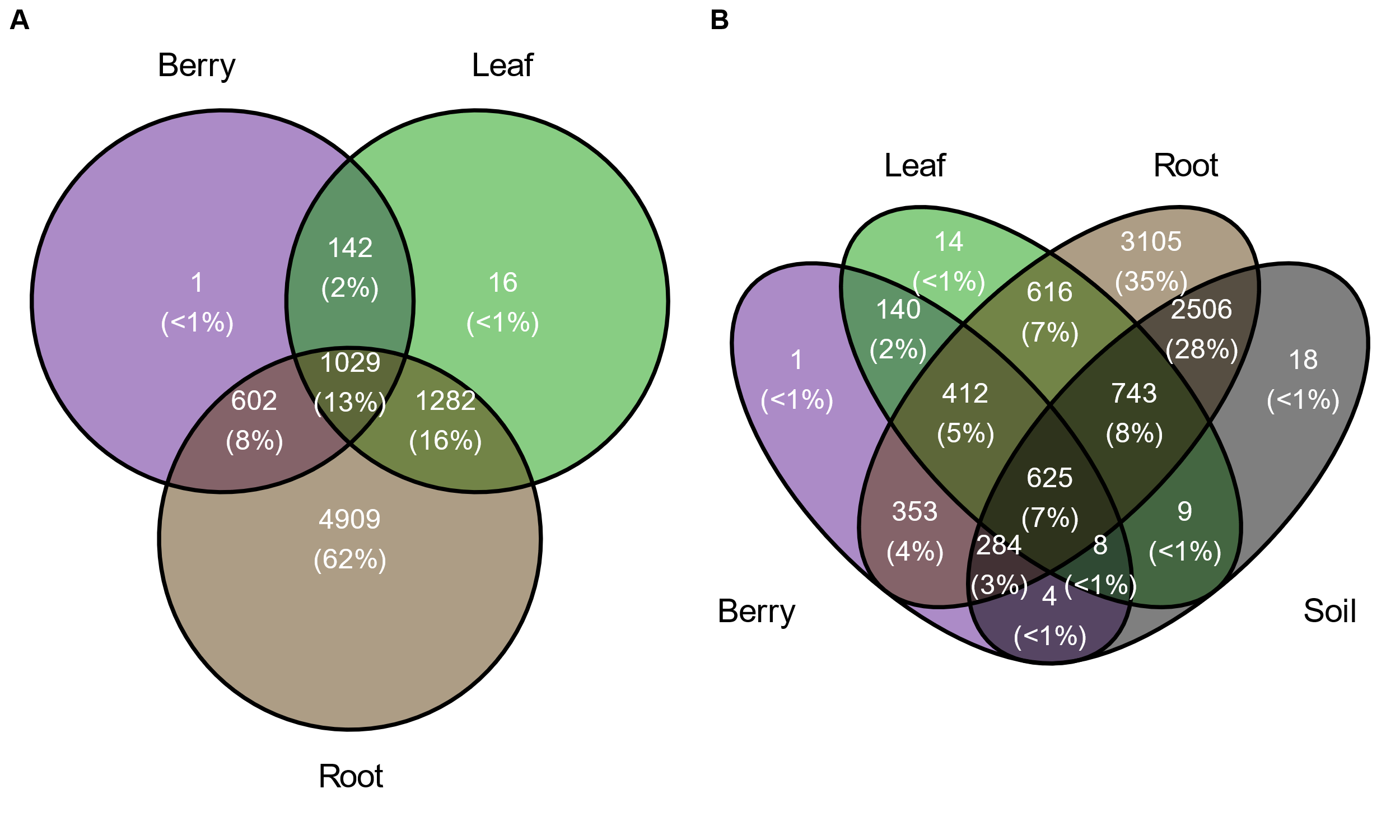


**Figure S6.** Principal coordinate analysis of Bray-Curtis dissimilarity for all compartments (berry, leaf, root, soil). Points are colored according to **A-B)** plant compartment or **C-D)** collection site.


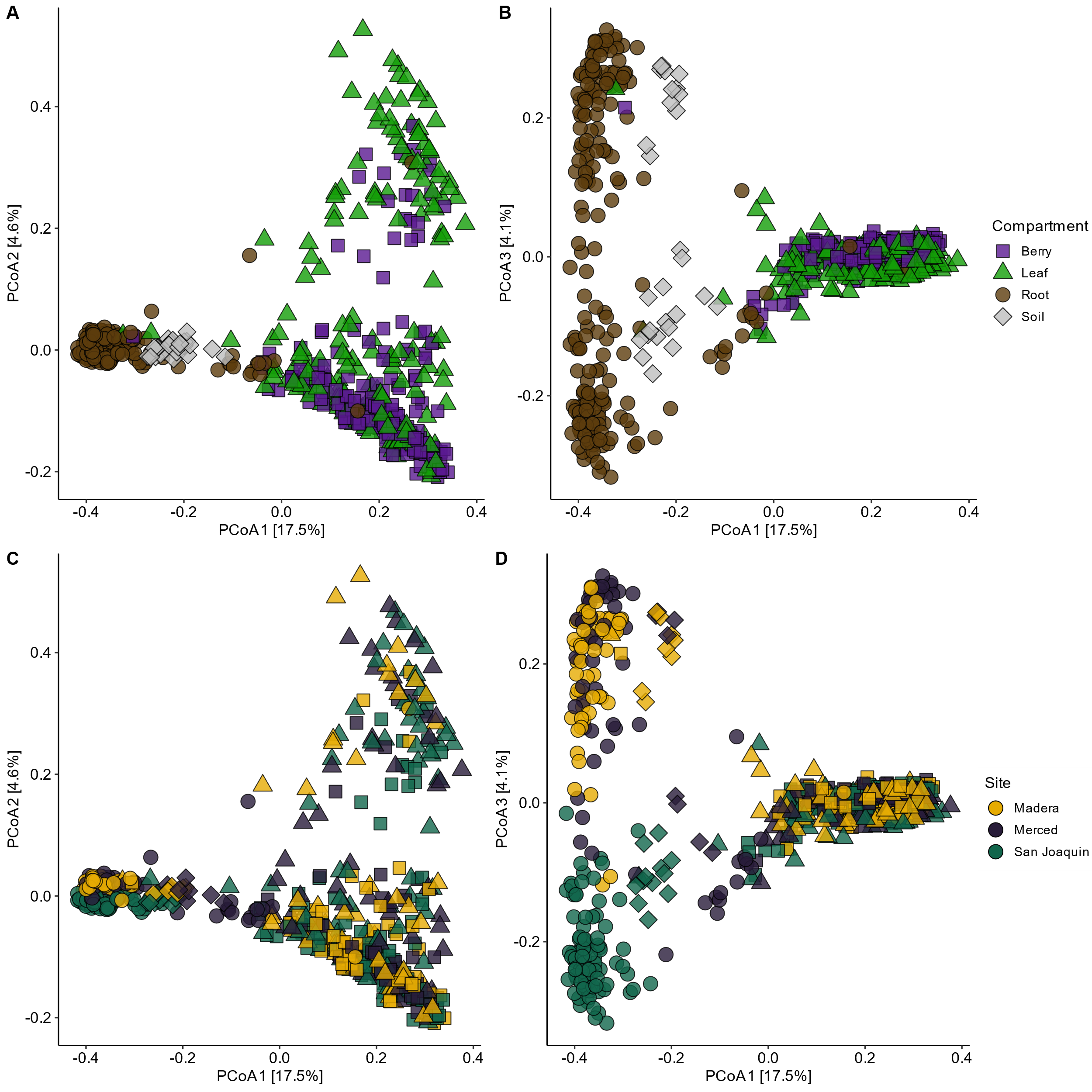


**Figure S7.** Principal coordinate analysis of Bray-Curtis dissimilarity for **A)** berry, **B)** leaf, and **C)** root samples. From left to right, points are colored according to rootstock genotype, scion genotype, collection year, collection site, and sugar content (measured from the berries of the vine at time of collection).


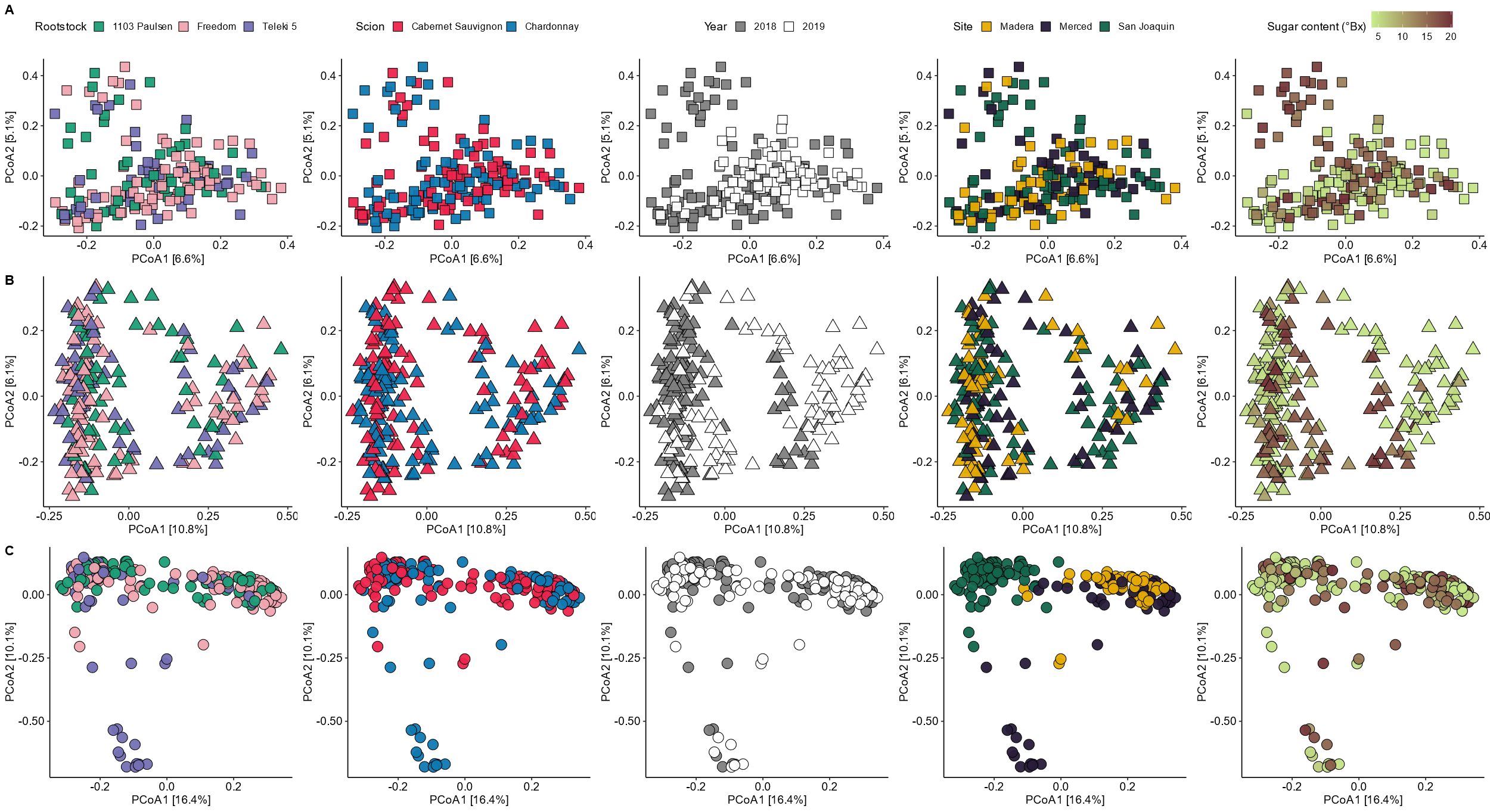


**Figure S8.** Confusion matrices for each experimental factor; A) plant compartment, B) Rootstock genotype, C) Scion genotype, D) Collection site, and E) Collection year. The model was trained using an 80:20 data split (80% train, 20% test) with 10-fold cross validation, shading represents the proportion of predictions for a label. Labels on the left represent the predicted labels and the labels on the bottom represent the actual labels. The model wide accuracy is given above each confusion matrix, class-wise statistics are provided in table S11-12.

**
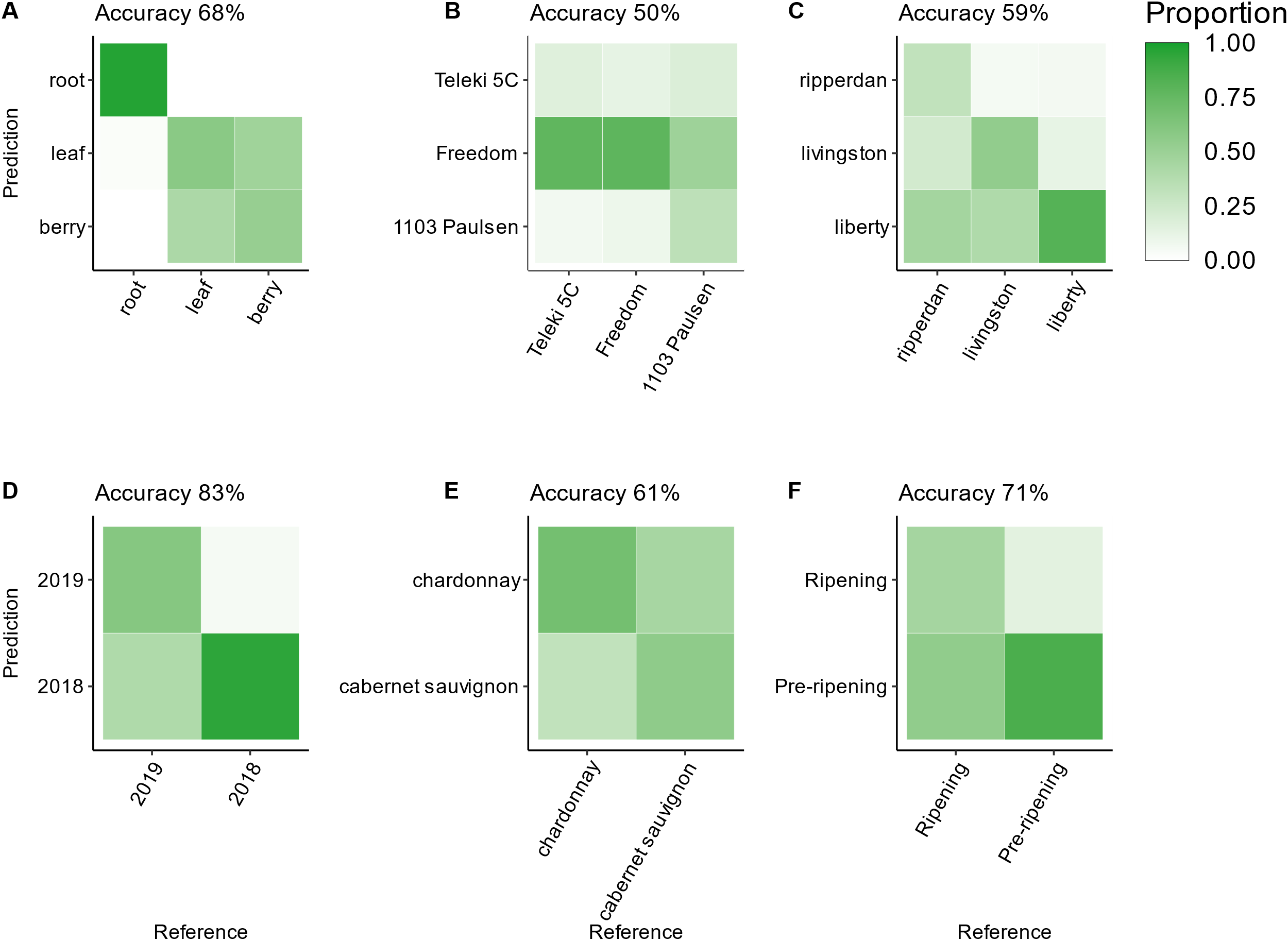
**

**Figure S9.** Relative importance of phyla to random forest classifiers for each experimental factor.


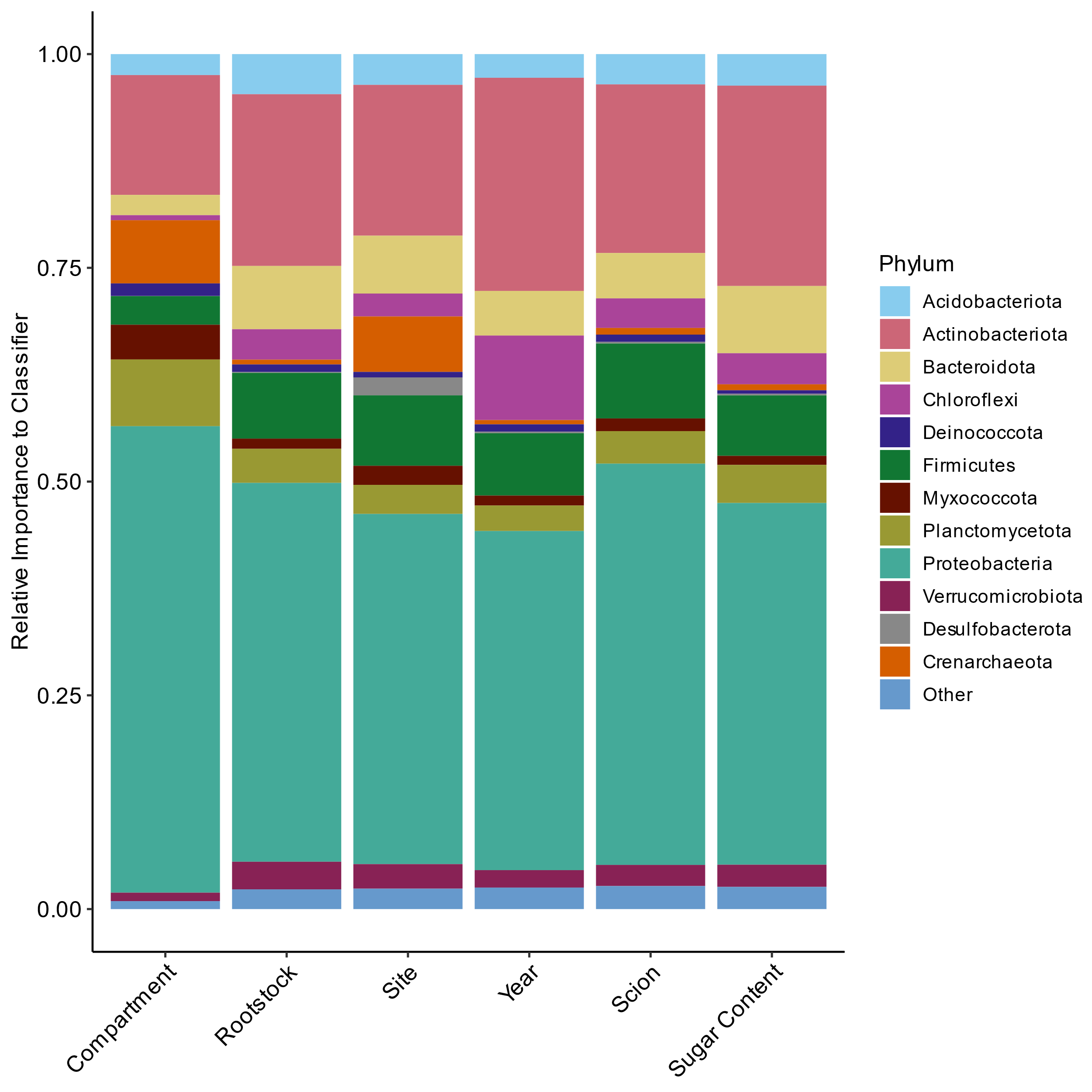


**Figure S10.** Individual ASVs that contribute to the random forest classifiers trained for; A) plant compartment, B) rootstock genotype, C) collection site, D) Collection year, E) scion genotype, and F) Sugar Content. Gini importance was assessed for all ASVs on the out-of-bag samples for each fold of the cross validation (*n*=10) and was scaled from 0 to 100 (higher being more important to the trained classifier). Only ASVs showed a greater than 25% decrease in gini importance are shown.

**
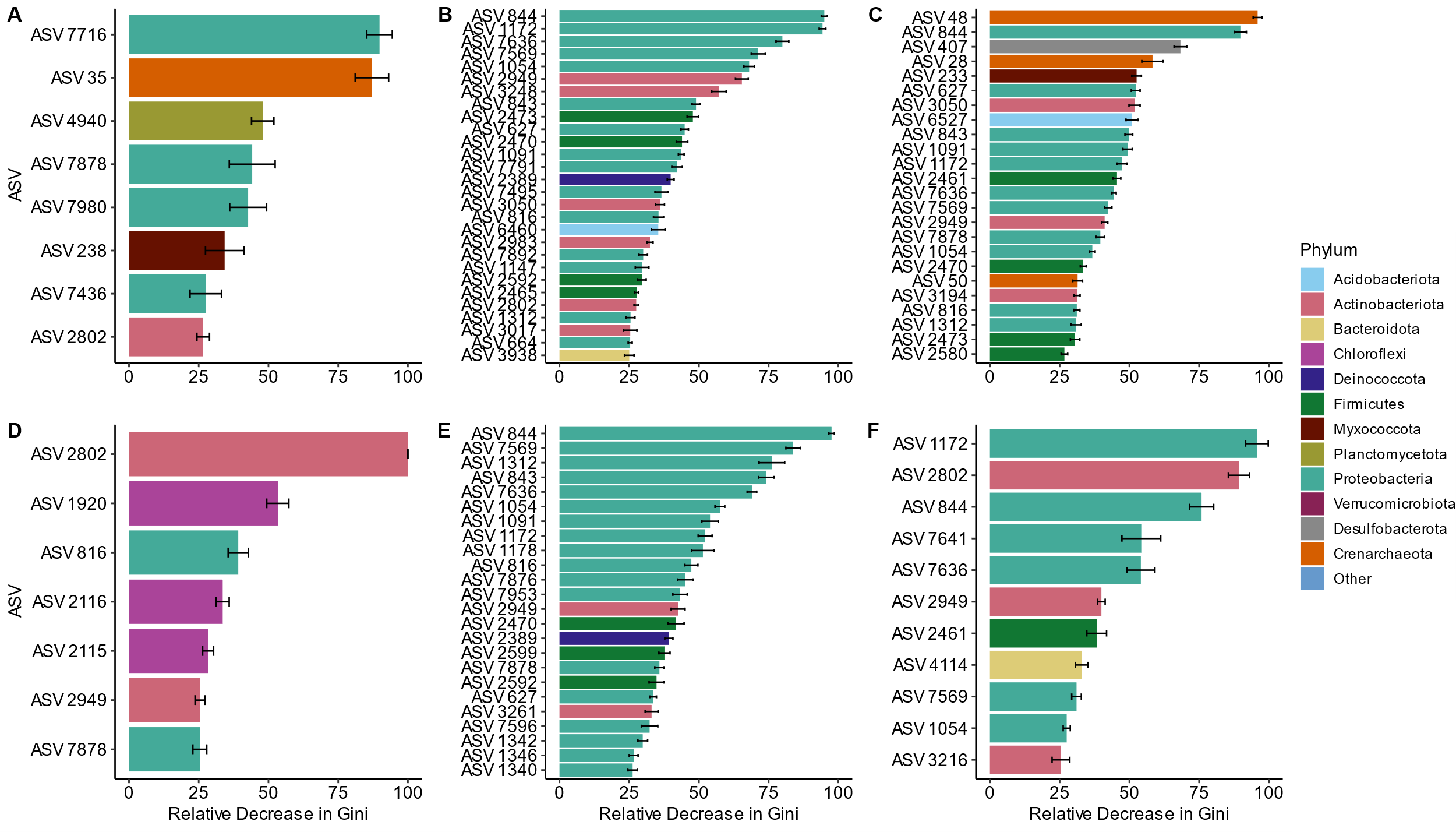
**

# References

1. Abatzoglou JT, Dobrowski SZ, Parks SA, Hegewisch KC. TerraClimate, a High-Resolution Global Dataset of Monthly Climate and Climatic Water Balance from 1958–2015. Sci Data. 2018**;***5*:170191. https://doi.org/10.1038/sdata.2017.191.
